# Author Correction: Generating synthetic task-based brain fingerprints for population neuroscience using deep learning

**DOI:** 10.1038/s42003-026-10937-y

**Published:** 2026-09-12

**Authors:** Emin Serin, Kerstin Ritter, Gunter Schumann, Tobias Banaschewski, Andre Marquand, Henrik Walter

**Affiliations:** 1 Research Division of Mind and Brain, Department of Psychiatry and Neuroscience CCM, Charité-Universitätsmedizin Berlin, Corporate Member of Freie Universität Berlin, Humboldt-Universität zu Berlin and Berlin Institute of Health, Berlin, Germany; 2 Einstein Center for Neurosciences Berlin, Charité–Universitätsmedizin Berlin, Corporate Member of Freie Universität Berlin and Humboldt-Universität zu Berlin and Berlin Institute of Health, Berlin, Germany; 3 Bernstein Center for Computational Neuroscience Berlin, Berlin, Germany; 4 Hertie Institute for AI in Brain Health, University of Tübingen, Tübingen, Germany; 5 Department of Psychiatry and Neuroscience CCM, Centre of Population Neuroscience and Stratified Medicine (PONS), Charité-Universitätsmedizin Berlin, Corporate Member of Freie Universität Berlin, Humboldt-Universität zu Berlin, and Berlin Institute of Health, Berlin, Germany; 6 Centre for Population Neuroscience and Stratified Medicine (PONS), Institute for Science and Technology of Brain-inspired Intelligence (ISTBI), Fudan University, Shanghai, China; 7 Department of Child and Adolescent Psychiatry and Psychotherapy, Central Institute of Mental Health, Medical Faculty Mannheim, University of Heidelberg, Mannheim, Germany; German Center for Mental Health, Mannheim, Heidelberg, Germany; 8 Donders Institute for Brain, Cognition and Behavior, Radboud University, Nijmegen, the Netherlands; 9 Department of Biochemistry CCM, Charité-Universitätsmedizin Berlin, Corporate Member of Freie Universität Berlin, Humboldt-Universität zu Berlin and Berlin Institute of Health, Berlin, Germany; 10 Berlin Institute of Health at Charité – Universitätsmedizin Berlin, Berlin, Germany; 11 Institute of Meteorology, Freie Universität Berlin, Berlin, Germany; 12 Institute of Medical Psychology and Medical Sociology, University Medical Center Schleswig-Holstein, Kiel, Germany; 13 Centre for Precision Psychiatry, Division of Mental Health and Addiction, Oslo University Hospital & Institute of Clinical Medicine, University of Oslo, Oslo, Norway; 14 Department of Psychology, University of Oslo, Oslo, Norway; 15 Social and Preventive Medicine, University of Potsdam, Potsdam, Germany; 16 Institute of Science and Technology Austria, Vienna, Austria; 17 Departament de Psicologia Clínica i Psicobiologia, Event Lab, Institute of Neuroscience of the University of Barcelona, Barcelona, Spain; 18 Institute of Human Genetics, University of Bonn School of Medicine and University Hospital Bonn, Bonn, Germany; 19 Ksilink, Strasbourg, France; 20 Aix-Marseille Université, Marseille, France; 21 Virtual Bodyworks, Barcelona, Spain; 22 Department of Earth Observation, Friedrich Schiller University Jena, Jena, Germany; 23 Institute of Science and Technology for Brain-Inspired Intelligence, Fudan University, Fudan, China; 24 Fudan University, Fudan, China; 25 Tri-institutional Center for Translational Research in Neuroimaging and Data Science, Georgia State University, Georgia Institute of Technology, and Emory University, Atlanta, GA USA; 26 Concentris Research Mamagement gmbh, Fürstenfeldbruck, Germany; 27 Stevens Neuroimaging & Informatics Institute, Keck School of Medicine, University of Southern California, Los Angeles, USA; 28 Geo, Google LLC, Mountain View, CA USA; 29 Social, Genetic and Developmental Psychiatry Centre, King’s College London, London, UK; 30 Psychological Medicine, King’s College London, South London & Maudsely NHS Foundation Trust, London, UK; 31 IUF Leibniz Research Institute for Environmental Medicine, Düsseldorf, Germany; 32 The Norwegian Mother, Father and Child Cohort Study, Norwegian Institute of Public Health, Oslo, Norway; 33 School of Computer Science, University of Nottingham, Nottingham, UK

**Keywords:** Computational neuroscience, Predictive markers

Correction to: *Communications Biology* https://doi.org/10.1038/s42003-025-09158-6. published online 14 November 2025

In the version of this article initially published, in the third term of Eq. (2), now reading “= *LR *+ *α *max(*LR*−*LC*+ *λ*,0),” “max” appeared originally as “min.” The equation is now updated in the HTML and PDF versions of the article.

