# Supplementary material for: Generating synthetic task-based brain fingerprints for population neuroscience using deep learning
Source: Commun Biol. 2025 Nov 14;8:1572. doi: 10.1038/s42003-025-09158-6 (PMC12618474; doi:10.1038/s42003-025-09158-6)
Supplement: Supplementary file 1 — Supplementary Figs. and Tables [file 42003_2025_9158_MOESM1_ESM.pdf]

Supplementary Materials for "Generating Synthetic Task-based Brain Fingerprints for Population Neuroscience Using Deep Learning"

Preprocessing

Human Connectome Project Development (HCP-D): Similarly, the minimally preprocessed volumetric resting state (e.g., rfMRI_REST_hp0_clean.nii.gz) and task-fMRI (e.g., tfMRI_GUESSING_PA_hp0_clean.nii.gz) images provided by the HCP were used in our study. The voxel-to-ROI matrices for each subject within the dataset were computed using the same ICA-based parcellation used in the training dataset. The task-based contrast maps were calculated using "nilearn.glm.first_level.FirstLevelModel" method from Nilearn[1]. Specifically, the first level model with a high-pass filter (0.01 Hz), "glover" hemodynamic response function, smoothing with 6 mm FWHM Gaussian kernel, and z-transformation was applied to minimally preprocessed task-based images. Nuisance correction was done using the Friston 24 motion parameters.

UK Biobank (UKB): We used the consortium-processed dataset, which was preprocessed using an FSL-based pipeline, which includes unwarping, motion correction, fieldmap correction, registration, normalization, automatic noise selection as implemented in ICA-AROMA[2], and smoothing using a 5 mm FWHM Gaussian kernel. Following preprocessing, the same ICA-based parcellation used in the previous analyses was utilized to extract general-purpose resting-state images and compute voxel-to-ROI time series. Z-transformed contrast maps for the Emotion task were computed using FSL. Subjects with a mean relative root mean square head motion greater than 0.5 mm were excluded.

Additional Performance Metrics

Dice AUC Score*:* The Dice coefficient[3] (also known as the F1 score) is a widely used metric in medical image segmentation that measures the voxel-wise overlap between predicted and actual task-based contrast activation patterns (i.e.,

thresholded contrast maps), thereby capturing the spatial distribution similarity between images. To compute the Dice coefficient, both predicted and actual contrast maps were thresholded with a series of activity thresholds to generate binary maps of activated voxels. For each threshold t, the Dice coefficient is calculated as:

$$Dice_t = \frac{2|Predicted\ Map_t \cap Actual\ Map_t|}{|Predicted\ Map_t| + |Actual\ Map_t|},$$

where $|Predicted\ Map_t|$ and $|Actual\ Map_t|$ are the number of activated voxels in the predicted and actual contrast maps, respectively, and $|Predicted\ Map_t \cap Actual\ Map_t|$ denotes the overlap between them. To account for variability in activation thresholds, Dice scores over a range of thresholds (5% to 50% with 5% interval) were integrated to create Area Under the Dice Curve (Dice AUC)[4].

Fingerprinting Score*:* Fingerprinting score, introduced by Finn et al.[5] is another metric used to evaluate a model's ability to predict subject-specific contrast maps. It is computed by correlating each subject's predicted task contrast with both their actual task contrast and the actual task contrasts of all other subjects. The score represents the fraction of subjects whose predicted contrast achieves the highest correlation with their own actual contrast (i.e., argmax), quantifying individual predictability. Similar to the discriminability score, we normalize the diagonality index by dividing it by reconstruction performance. A discriminability score of 1 indicates perfect similarity to a subject's own actual contrast map and perfect separation from other subjects, suggesting that synthetic maps capture individual-specific characteristics beyond average information.

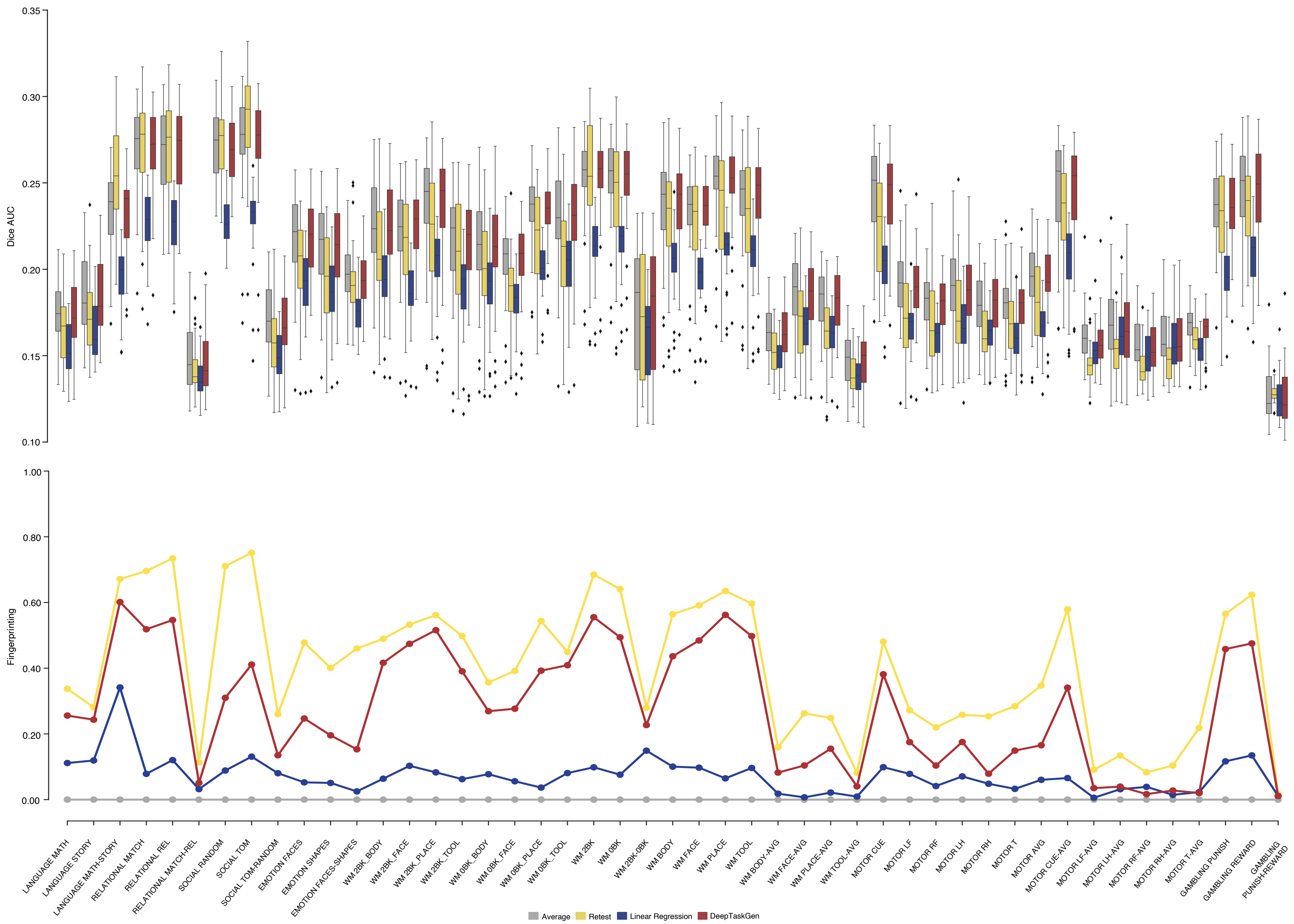
Dice AUC
0.35
0.30
0.25
0.20
0.15
0.10
Fingerprinting
1.00
0.80
0.60
0.40
0.20
0.00
LANGUAGE MATH
LANGUAGE STORY
LANGUAGE MATH-STORY
RELATIONAL MATCH
RELATIONAL REL
RELATIONAL MATCH-REL
SOCIAL RANDOM
SOCIAL TOM
SOCIAL TOM-RANDOM
EMOTION FACES
EMOTION SHAPES
EMOTION FACES-SHAPES
WM 2BK_BODY
WM 2BK_FACE
WM 2BK_PLACE
WM 2BK_TOOL
WM 0BK_BODY
WM 0BK_FACE
WM 0BK_PLACE
WM 0BK_TOOL
WM 2BK
WM 0BK
WM 2BK-0BK
WM BODY
WM FACE
WM PLACE
WM TOOL
WM BODY-AVG
WM FACE-AVG
WM PLACE-AVG
WM TOOL-AVG
MOTOR CUE
MOTOR LF
MOTOR RF
MOTOR LH
MOTOR RH
MOTOR T
MOTOR AVG
MOTOR CUE-AVG
MOTOR LF-AVG
MOTOR LH-AVG
MOTOR RF-AVG
MOTOR RH-AVG
MOTOR T-AVG
GAMBLING PUNISH
GAMBLING REWARD
GAMBLING PUNISH-REWARD
Average
Retest
Linear Regression
DeepTaskGen

Supplementary Figure 1. Dice AUC and fingerprinting score of DeepTaskGen and various baselines for 47 task contrasts from HCP-YA. In boxplots, the box ranges from the first quartile to the third quartile, with a line inside indicating the median. The "whiskers" extend to the most extreme values within 1.5 times the interquartile range, which are not considered outliers. Any points outside this range are plotted individually as outliers.

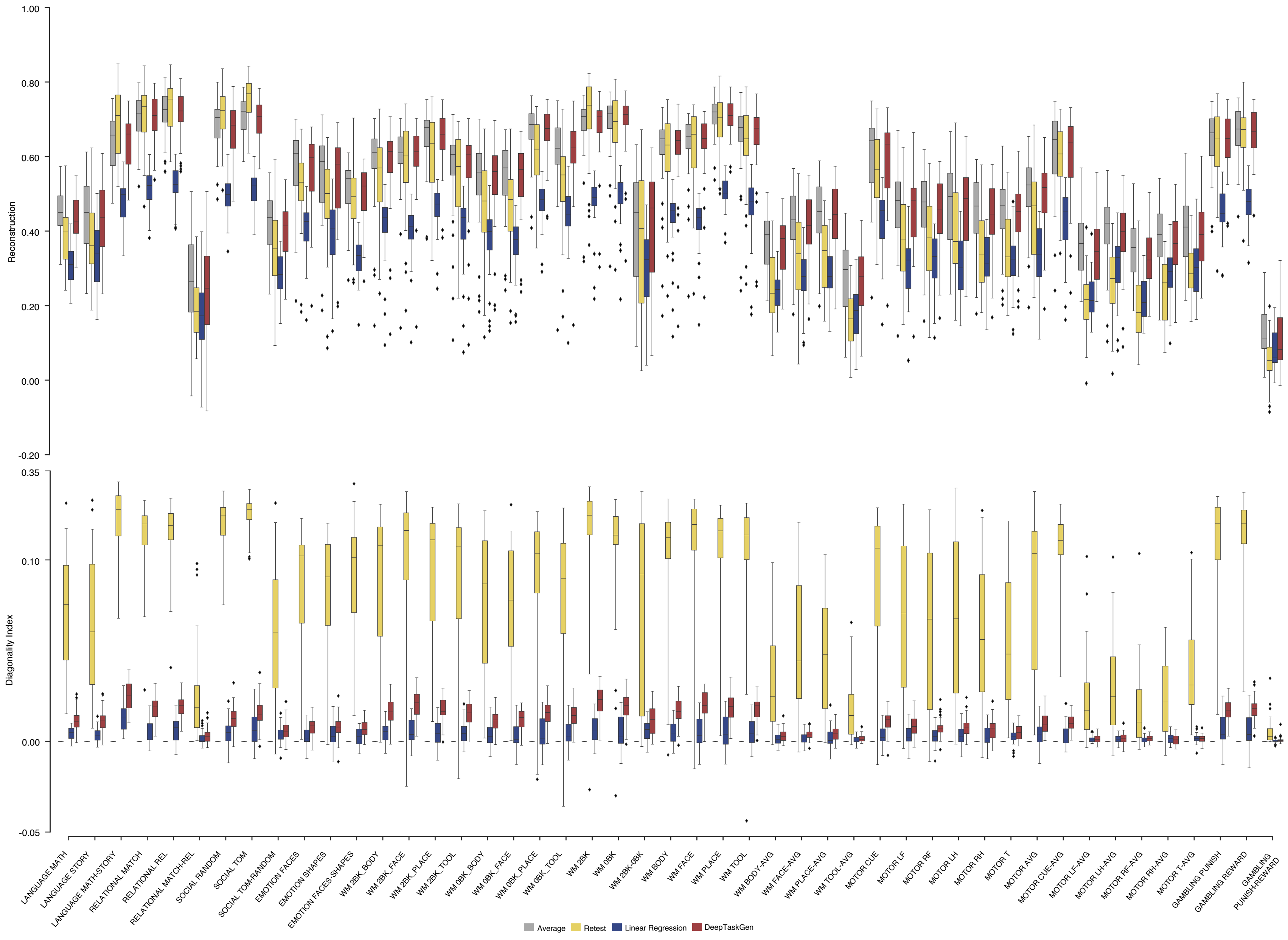

Reconstruction
1.00
0.80
0.60
0.40
0.20
0.00
-0.20
Diagonality Index
0.35
0.10
0.00
-0.05
LANGUAGE MATH
LANGUAGE STORY
LANGUAGE MATH-STORY
RELATIONAL MATCH
RELATIONAL REL
RELATIONAL MATCH-REL
SOCIAL RANDOM
SOCIAL TOM
SOCIAL TOM-RANDOM
EMOTION FACES
EMOTION SHAPES
EMOTION FACES-SHAPES
WM 2BK_BODY
WM 2BK_FACE
WM 2BK_PLACE
WM 2BK_TOOL
WM 0BK_BODY
WM 0BK_FACE
WM 0BK_PLACE
WM 0BK_TOOL
WM 2BK
WM 0BK
WM 2BK-0BK
WM BODY
WM FACE
WM PLACE
WM TOOL
WM BODY-AVG
WM FACE-AVG
WM PLACE-AVG
WM TOOL-AVG
MOTOR CUE
MOTOR LF
MOTOR RF
MOTOR LH
MOTOR RH
MOTOR T
MOTOR AVG
MOTOR CUE-AVG
MOTOR LF-AVG
MOTOR LH-AVG
MOTOR RF-AVG
MOTOR RH-AVG
MOTOR T-AVG
GAMBLING PUNISH
GAMBLING REWARD
GAMBLING PUNISH-REWARD
Average
Retest
Linear Regression
DeepTaskGen

Supplementary Figure 2. Reconstruction performance computed by taking Pearson's correlation between predicted and actual contrast maps and the diagonality index (the difference between the on-diagonal and the mean off-diagonal elements in a correlation matrix, normalized by the mean on-diagonal values) of DeepTaskGen and various baselines for 47 task contrasts from HCP-YA. The diagonality index scores were given in a symmetrical log scale (symlog, threshold = 0.10). In boxplots, the box ranges from the first quartile to the third quartile, with a line inside indicating the median. The "whiskers" extend to the most extreme values within 1.5 times the interquartile range, which are not considered outliers. Any points outside this range are plotted individually as outliers.

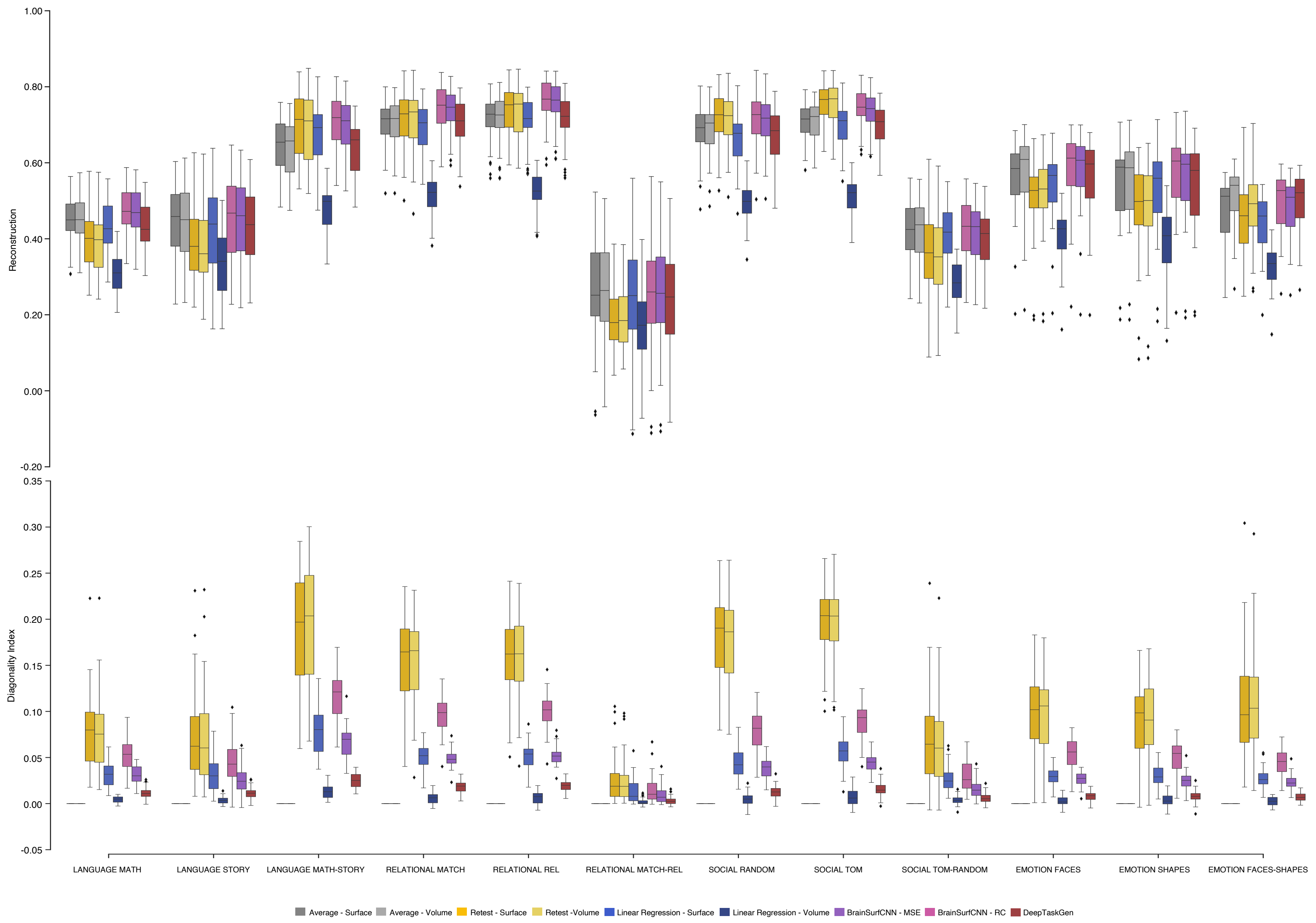

Reconstruction
Diagonality Index
LANGUAGE MATH
LANGUAGE STORY
LANGUAGE MATH-STORY
RELATIONAL MATCH
RELATIONAL REL
RELATIONAL MATCH-REL
SOCIAL RANDOM
SOCIAL TOM
SOCIAL TOM-RANDOM
EMOTION FACES
EMOTION SHAPES
EMOTION FACES-SHAPES
Average - Surface
Average - Volume
Retest - Surface
Retest -Volume
Linear Regression - Surface
Linear Regression - Volume
BrainSurfCNN - MSE
BrainSurfCNN - RC
DeepTaskGen

Supplementary Figure 3. Reconstruction performance and diagonality index for surface- and volume-based methods, as well as baselines for task contrasts from the LANGUAGE, RELATIONAL, SOCIAL, and EMOTION tasks in the HCP-YA dataset. Average: Group Average; Retest: Test-Retest subjects; Linear Regression: Tavor et al., 2016[6]; BrainSurfCNN - MSE: 50 epochs with MSE loss (i.e., first training); BrainSurfCNN - RC: Final model presented in Ngo et al., 2022[4]: Fine-tuned model with 50 epochs using reconstructive-contrastive loss; DeepTaskGen - proposed method. In boxplots, the box ranges from the first quartile to the third quartile, with a line inside indicating the median. The "whiskers" extend to the most extreme values within 1.5 times the interquartile range, which are not considered outliers. Any points outside this range are plotted individually as outliers.

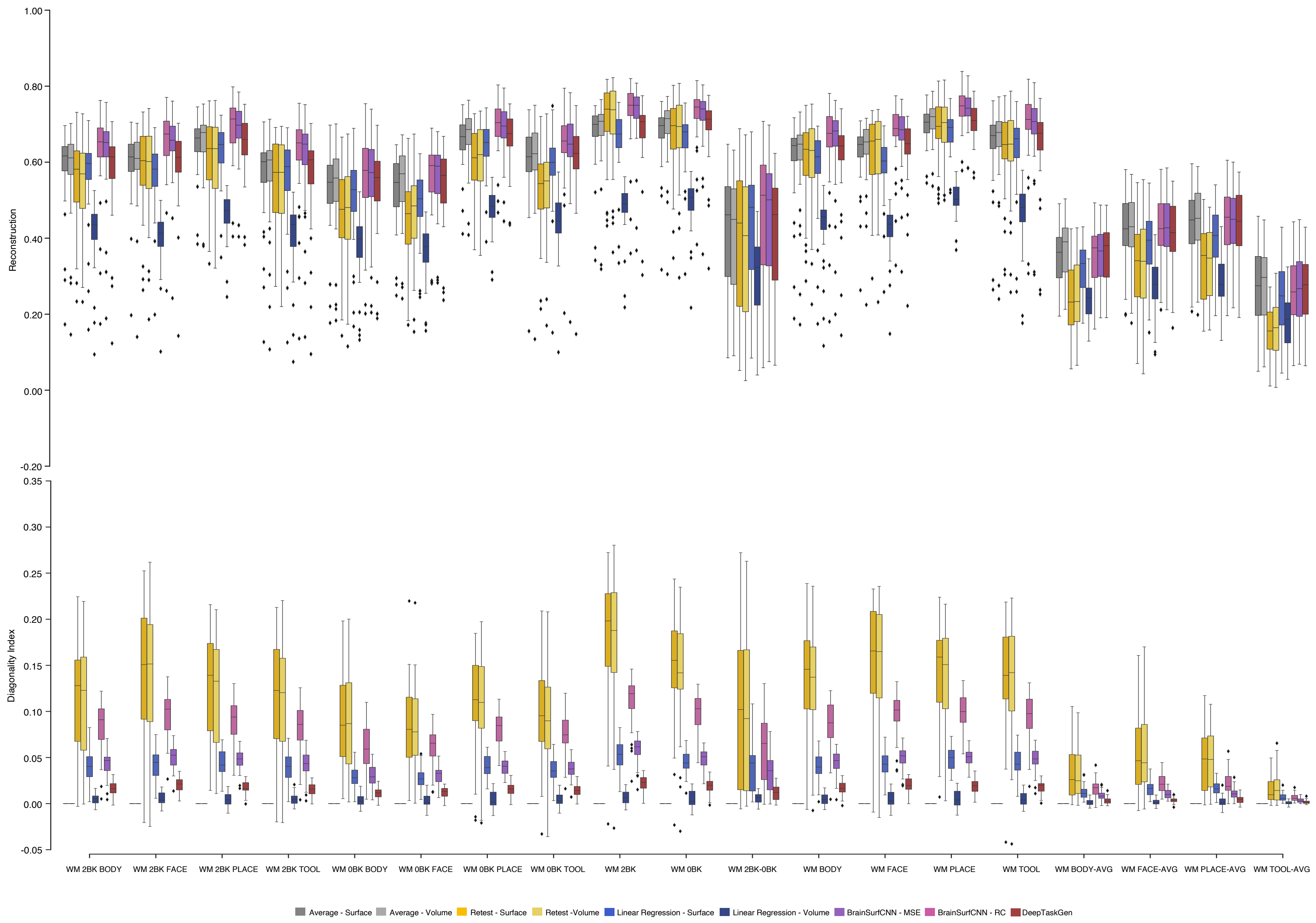

Reconstruction
1.00
0.80
0.60
0.40
0.20
0.00
-0.20
Diagonality Index
0.35
0.30
0.25
0.20
0.15
0.10
0.05
0.00
-0.05
WM 2BK BODY
WM 2BK FACE
WM 2BK PLACE
WM 2BK TOOL
WM 0BK BODY
WM 0BK FACE
WM 0BK PLACE
WM 0BK TOOL
WM 2BK
WM 0BK
WM 2BK-0BK
WM BODY
WM FACE
WM PLACE
WM TOOL
WM BODY-AVG
WM FACE-AVG
WM PLACE-AVG
WM TOOL-AVG
Average - Surface
Average - Volume
Retest - Surface
Retest -Volume
Linear Regression - Surface
Linear Regression - Volume
BrainSurfCNN - MSE
BrainSurfCNN - RC
DeepTaskGen

Supplementary Figure 4. Reconstruction performance and diagonality index for surface- and volume-based methods, as well as baselines for task contrasts from the WM task in the HCP-YA dataset. Average: Group Average; Retest: Test-Retest subjects; Linear Regression: Tavor et al., 2016[6]; BrainSurfCNN - MSE: 50 epochs with MSE loss (i.e., first training); BrainSurfCNN - RC: Final model presented in Ngo et al., 2022[4]; DeepTaskGen - proposed method. In boxplots, the box ranges from the first quartile to the third quartile, with a line inside indicating the median. The "whiskers" extend to the most extreme values within 1.5 times the interquartile range, which are not considered outliers. Any points outside this range are plotted individually as outliers.

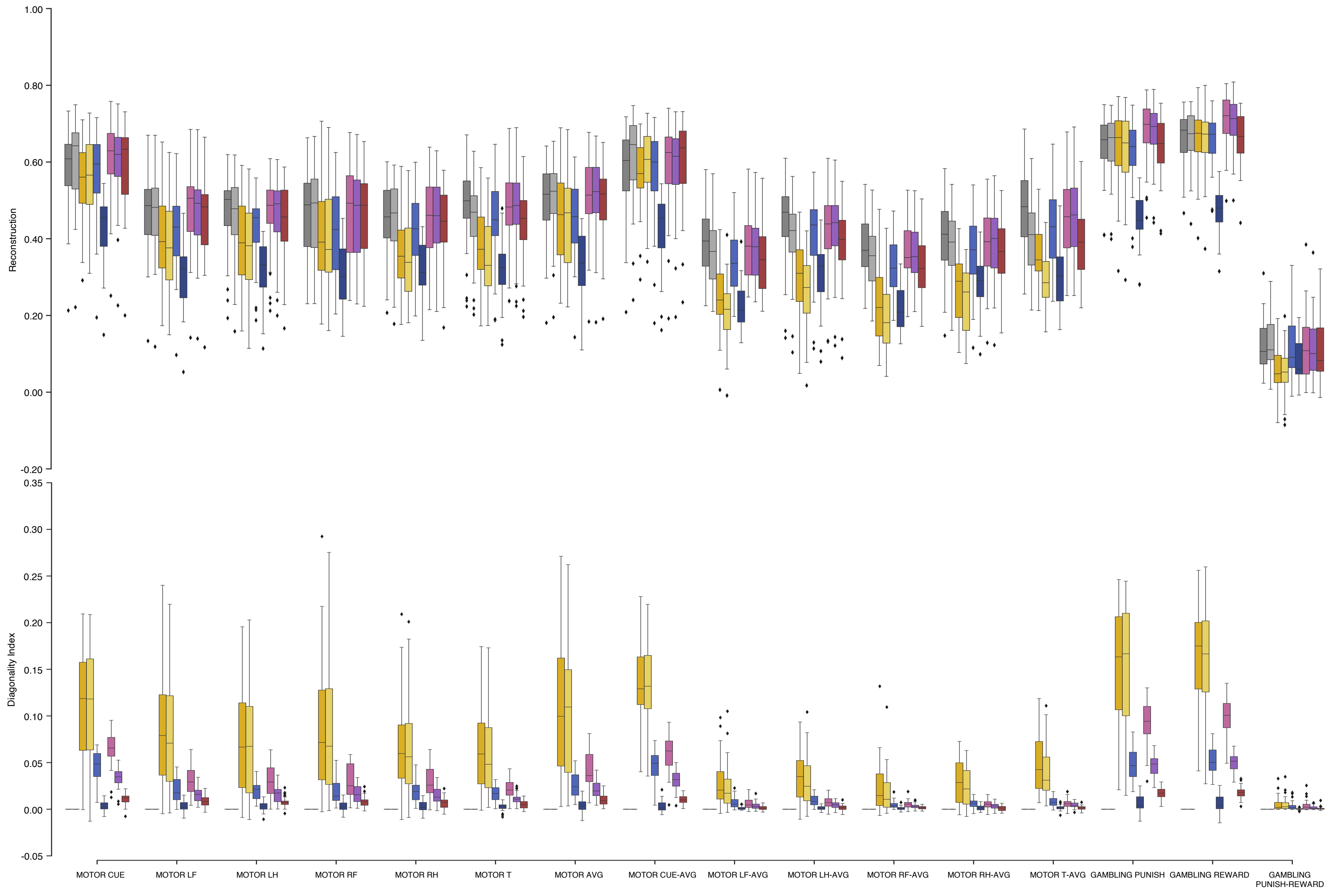

Reconstruction
1.00
0.80
0.60
0.40
0.20
0.00
-0.20
Diagonality Index
0.35
0.30
0.25
0.20
0.15
0.10
0.05
0.00
-0.05
MOTOR CUE
MOTOR LF
MOTOR LH
MOTOR RF
MOTOR RH
MOTOR T
MOTOR AVG
MOTOR CUE-AVG
MOTOR LF-AVG
MOTOR LH-AVG
MOTOR RF-AVG
MOTOR RH-AVG
MOTOR T-AVG
GAMBLING PUNISH
GAMBLING REWARD
GAMBLING PUNISH-REWARD
Average - Surface
Average - Volume
Retest - Surface
Retest -Volume
Linear Regression - Surface
Linear Regression - Volume
BrainSurfCNN - MSE
BrainSurfCNN - RC
DeepTaskGen

Supplementary Figure 5. Reconstruction performance and diagonality index for surface- and volume-based methods, as well as baselines for task contrasts from the MOTOR, and GAMBLING tasks in the HCP-YA dataset. Average: Group Average; Retest: Test-Retest subjects; Linear Regression: Tavor et al., 2016[6]; BrainSurfCNN - MSE: 50 epochs with MSE loss (i.e., first training); BrainSurfCNN - RC: Final model presented in Ngo et al., 2022[4]; DeepTaskGen - proposed method. In boxplots, the box ranges from the first quartile to the third quartile, with a line inside indicating the median. The "whiskers" extend to the most extreme values within 1.5 times the interquartile range, which are not considered outliers. Any points outside this range are plotted individually as outliers.

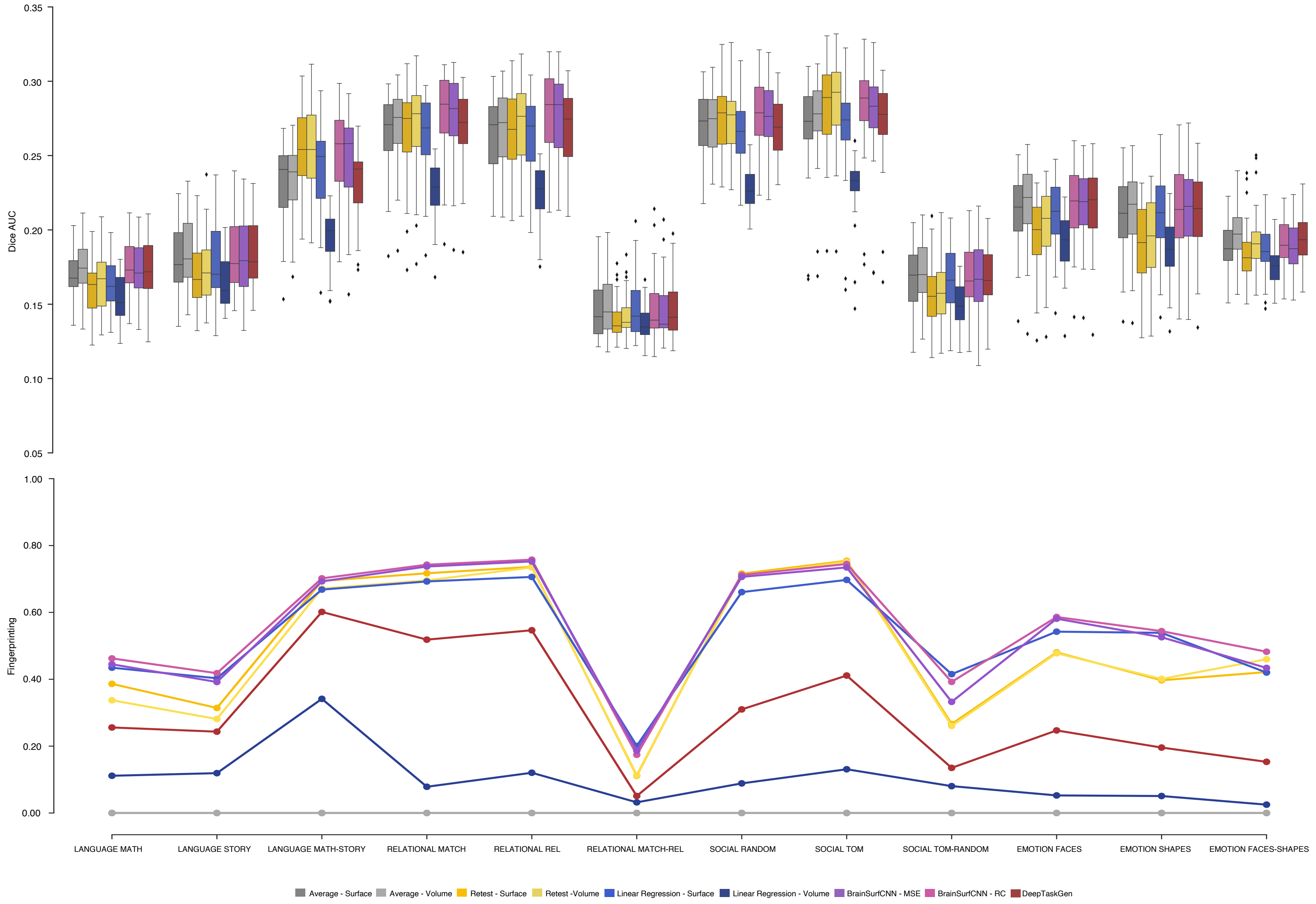

Dice AUC
0.35
0.30
0.25
0.20
0.15
0.10
0.05
Fingerprinting
1.00
0.80
0.60
0.40
0.20
0.00
LANGUAGE MATH
LANGUAGE STORY
LANGUAGE MATH-STORY
RELATIONAL MATCH
RELATIONAL REL
RELATIONAL MATCH-REL
SOCIAL RANDOM
SOCIAL TOM
SOCIAL TOM-RANDOM
EMOTION FACES
EMOTION SHAPES
EMOTION FACES-SHAPES
Average - Surface
Average - Volume
Retest - Surface
Retest -Volume
Linear Regression - Surface
Linear Regression - Volume
BrainSurfCNN - MSE
BrainSurfCNN - RC
DeepTaskGen

Supplementary Figure 6. Dice AUC and fingerprinting scores for surface- and volume-based methods, as well as baselines for task contrasts from the LANGUAGE, RELATIONAL, SOCIAL, and EMOTION tasks in the HCP-YA dataset. Average: Group Average; Retest: Test-Retest subjects; Linear Regression: Tavor et al., 2016[6]; BrainSurfCNN - MSE: 50 epochs with MSE loss (i.e., first training); BrainSurfCNN - RC: Final model presented in Ngo et al., 2022[4]; DeepTaskGen - proposed method. In boxplots, the box ranges from the first quartile to the third quartile, with a line inside indicating the median. The "whiskers" extend to the most extreme values within 1.5 times the interquartile range, which are not considered outliers. Any points outside this range are plotted individually as outliers.

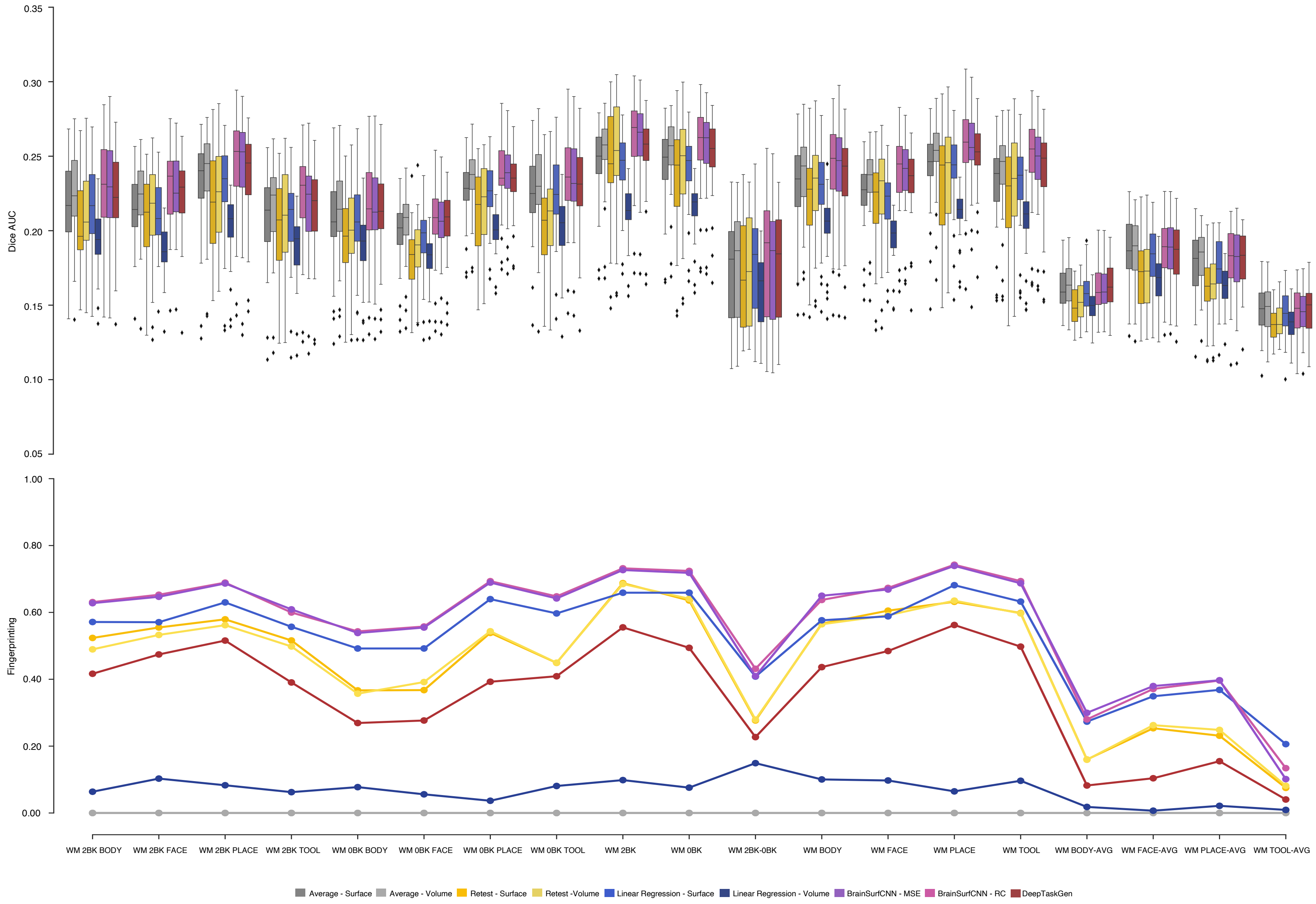
Dice AUC
0.35
0.30
0.25
0.20
0.15
0.10
0.05
Fingerprinting
1.00
0.80
0.60
0.40
0.20
0.00
WM 2BK BODY
WM 2BK FACE
WM 2BK PLACE
WM 2BK TOOL
WM 0BK BODY
WM 0BK FACE
WM 0BK PLACE
WM 0BK TOOL
WM 2BK
WM 0BK
WM 2BK-0BK
WM BODY
WM FACE
WM PLACE
WM TOOL
WM BODY-AVG
WM FACE-AVG
WM PLACE-AVG
WM TOOL-AVG
Average - Surface
Average - Volume
Retest - Surface
Retest -Volume
Linear Regression - Surface
Linear Regression - Volume
BrainSurfCNN - MSE
BrainSurfCNN - RC
DeepTaskGen

Supplementary Figure 7. Dice AUC and fingerprinting scores for surface- and volume-based methods, as well as baselines for task contrasts from the WM task in the HCP-YA dataset. Average: Group Average; Retest: Test-Retest subjects; Linear Regression: Tavor et al., 2016[6]; BrainSurfCNN - MSE: 50 epochs with MSE loss (i.e., first training); BrainSurfCNN - RC: Final model presented in Ngo et al., 2022[4]; DeepTaskGen - proposed method. In boxplots, the box ranges from the first quartile to the third quartile, with a line inside indicating the median. The "whiskers" extend to the most extreme values within 1.5 times the interquartile range, which are not considered outliers. Any points outside this range are plotted individually as outliers.

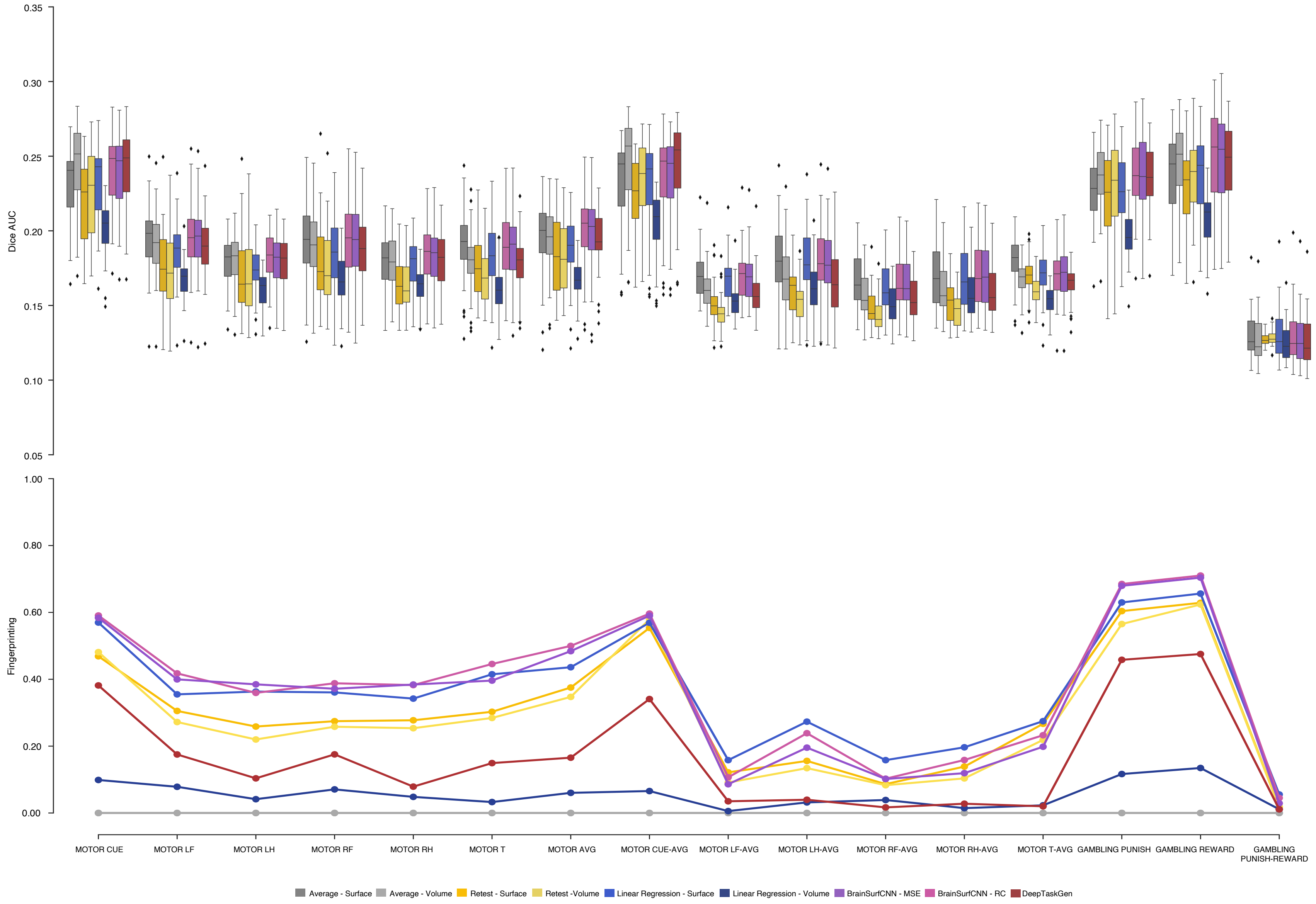

Dice AUC
0.35
0.30
0.25
0.20
0.15
0.10
0.05
Fingerprinting
1.00
0.80
0.60
0.40
0.20
0.00
MOTOR CUE
MOTOR LF
MOTOR LH
MOTOR RF
MOTOR RH
MOTOR T
MOTOR AVG
MOTOR CUE-AVG
MOTOR LF-AVG
MOTOR LH-AVG
MOTOR RF-AVG
MOTOR RH-AVG
MOTOR T-AVG
GAMBLING PUNISH
GAMBLING REWARD
GAMBLING PUNISH-REWARD
Average - Surface
Average - Volume
Retest - Surface
Retest -Volume
Linear Regression - Surface
Linear Regression - Volume
BrainSurfCNN - MSE
BrainSurfCNN - RC
DeepTaskGen

Supplementary Figure 8. Dice AUC and fingerprinting scores for surface- and volume-based methods, as well as baselines for task contrasts from the MOTOR and GAMBLING tasks in the HCP-YA dataset. Average: Group Average; Retest: Test-Retest subjects; Linear Regression: Tavor et al., 2016[6]; BrainSurfCNN - MSE: 50 epochs with MSE loss (i.e., first training); BrainSurfCNN - RC: Final model presented in Ngo et al., 2022[4]; DeepTaskGen - proposed method. In boxplots, the box ranges from the first quartile to the third quartile, with a line inside indicating the median. The "whiskers" extend to the most extreme values within 1.5 times the interquartile range, which are not considered outliers. Any points outside this range are plotted individually as outliers.

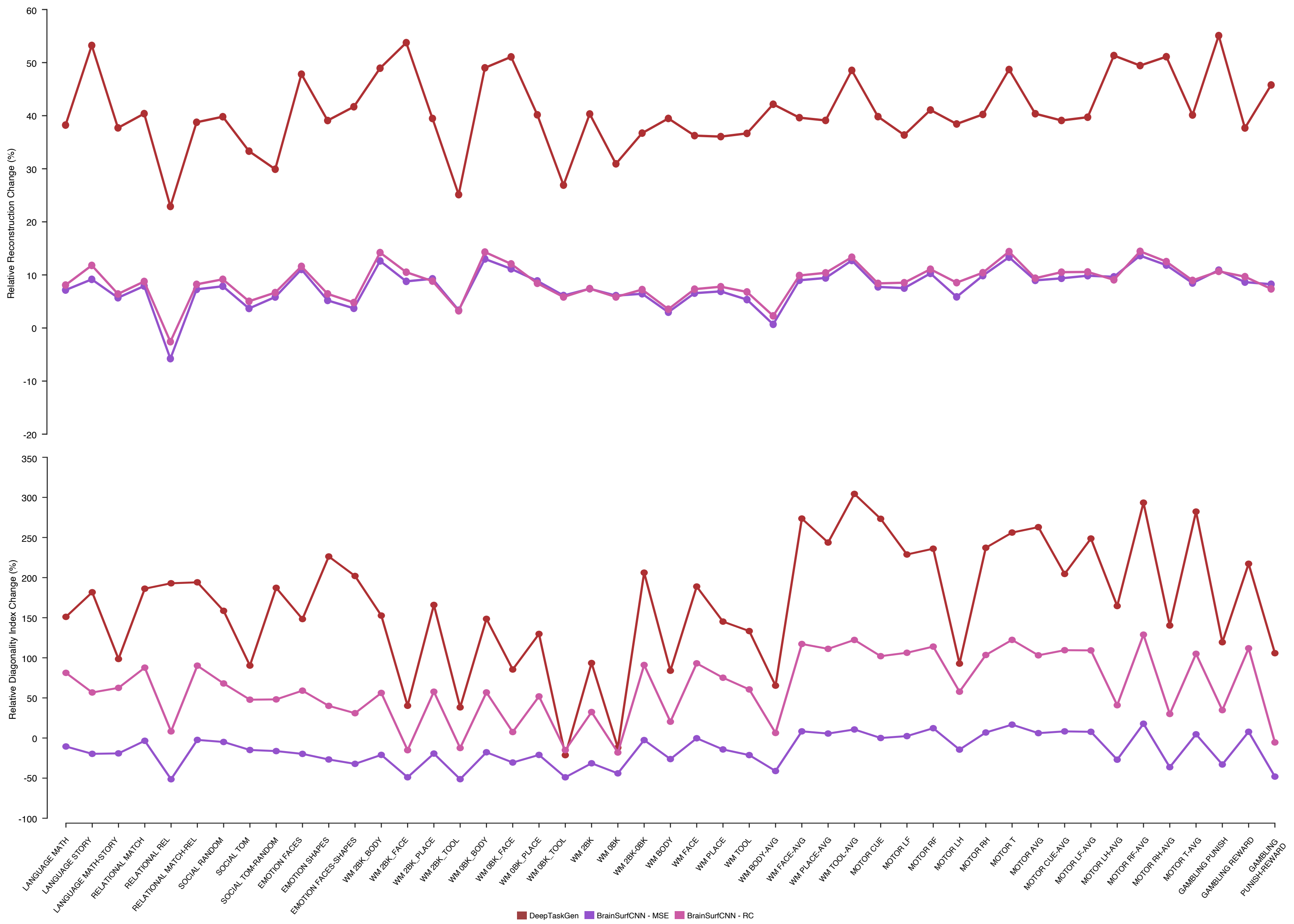

Relative Reconstruction Change (%)
60
50
40
30
20
10
0
-10
-20
Relative Diagonality Index Change (%)
350
300
250
200
150
100
50
0
-50
-100
LANGUAGE MATH
LANGUAGE STORY
LANGUAGE MATH-STORY
RELATIONAL MATCH
RELATIONAL REL
RELATIONAL MATCH-REL
SOCIAL RANDOM
SOCIAL TOM
SOCIAL TOM-RANDOM
EMOTION FACES
EMOTION SHAPES
EMOTION FACES-SHAPES
WM 2BK_BODY
WM 2BK_FACE
WM 2BK_PLACE
WM 2BK_TOOL
WM 0BK_BODY
WM 0BK_FACE
WM 0BK_PLACE
WM 0BK_TOOL
WM 2BK
WM 0BK
WM 2BK-0BK
WM BODY
WM FACE
WM PLACE
WM TOOL
WM BODY-AVG
WM FACE-AVG
WM PLACE-AVG
WM TOOL-AVG
MOTOR CUE
MOTOR LF
MOTOR RF
MOTOR LH
MOTOR RH
MOTOR T
MOTOR AVG
MOTOR CUE-AVG
MOTOR LF-AVG
MOTOR LH-AVG
MOTOR RF-AVG
MOTOR RH-AVG
MOTOR T-AVG
GAMBLING PUNISH
GAMBLING REWARD
GAMBLING PUNISH-REWARD
DeepTaskGen
BrainSurfCNN - MSE
BrainSurfCNN - RC

Supplementary Figure 9. Relative reconstruction performance and diagonality index scores of BrainSurfCNN and DeepTaskGen are shown for 47 task contrasts from HCP-YA. Relative scores are computed against the corresponding baseline linear regression[6] model's performance, indicating the models' performance gain or loss compared to the baseline linear model (i.e., $Relative\ Performance\ =\ ((Model\ Performance\ -\ Baseline\ Performance)\ /\ Baseline\ Performance) * 100$). Positive values indicate performance gains, and negative values indicate losses relative to the baseline. Model variants include: DeepTaskGen (proposed method); BrainSurfCNN - MSE: 50 epochs with MSE loss (i.e., first training); BrainSurfCNN - RC: Final model presented in Ngo et al., 2022[4]. Raw performance values are provided in Supplementary Figs. 3-5. DeepTaskGen achieves greater gains over the baseline than the surface-based BrainSurfCNN. Despite differences in magnitude, all models show a similar pattern of gains and losses across the 47 contrasts, with aligned peaks and dips, reflecting variability across task contrast maps.

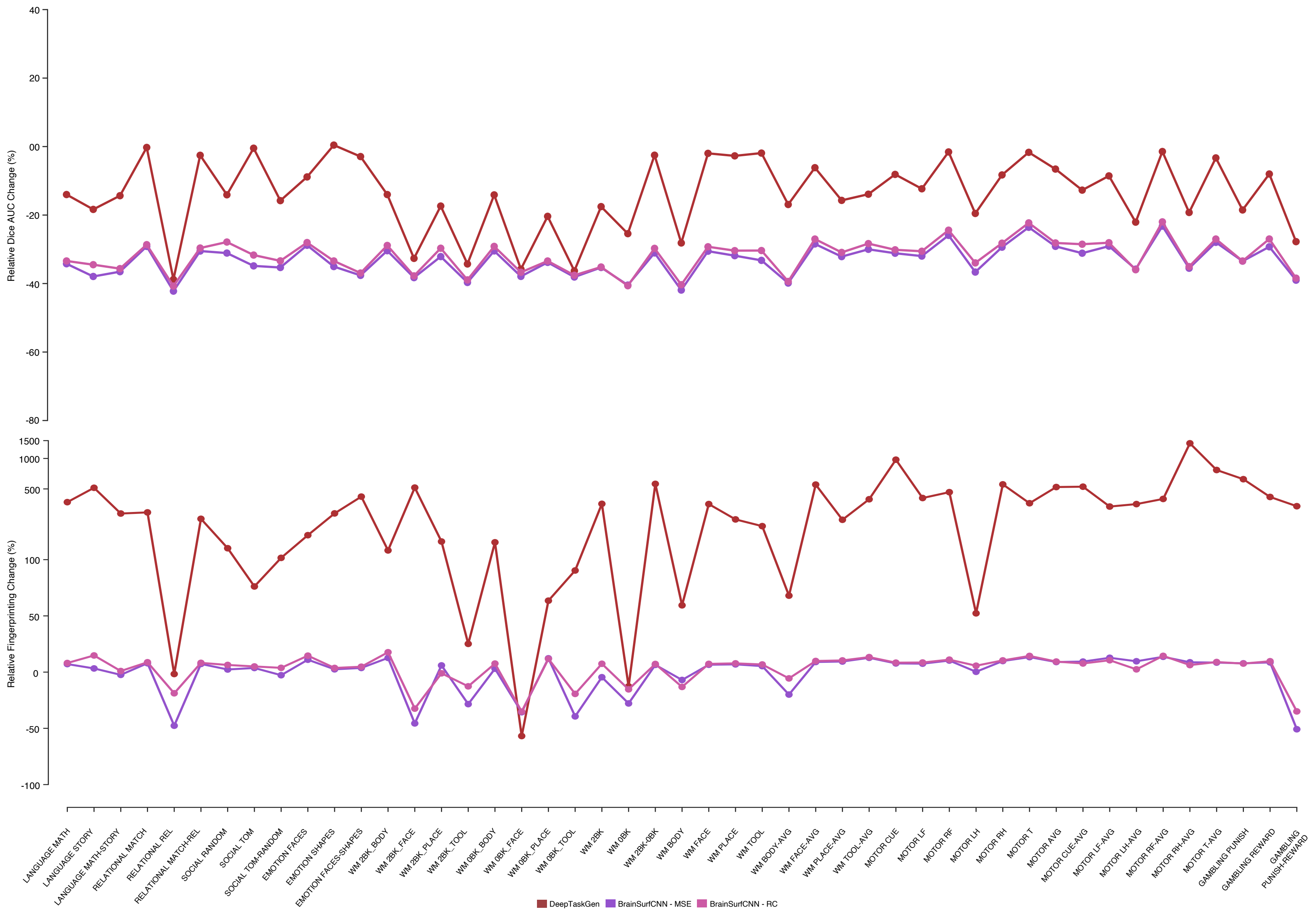
40
20
00
-20
-40
-60
-80
Relative Dice AUC Change (%)
1500
1000
500
100
50
0
-50
-100
Relative Fingerprinting Change (%)
LANGUAGE MATH
LANGUAGE STORY
LANGUAGE MATH-STORY
RELATIONAL MATCH
RELATIONAL REL
RELATIONAL MATCH-REL
SOCIAL RANDOM
SOCIAL TOM
SOCIAL TOM-RANDOM
EMOTION FACES
EMOTION SHAPES
EMOTION FACES-SHAPES
WM 2BK_BODY
WM 2BK_FACE
WM 2BK_PLACE
WM 2BK_TOOL
WM 0BK_BODY
WM 0BK_FACE
WM 0BK_PLACE
WM 0BK_TOOL
WM 2BK
WM 0BK
WM 2BK-0BK
WM BODY
WM FACE
WM PLACE
WM TOOL
WM BODY-AVG
WM FACE-AVG
WM PLACE-AVG
WM TOOL-AVG
MOTOR CUE
MOTOR LF
MOTOR RF
MOTOR LH
MOTOR RH
MOTOR T
MOTOR AVG
MOTOR CUE-AVG
MOTOR LF-AVG
MOTOR LH-AVG
MOTOR RF-AVG
MOTOR RH-AVG
MOTOR T-AVG
GAMBLING PUNISH
GAMBLING REWARD
GAMBLING PUNISH-REWARD
DeepTaskGen
BrainSurfCNN - MSE
BrainSurfCNN - RC

Supplementary Figure 10. Relative Dice AUC and fingerprinting scores of BrainSurfCNN and DeepTaskGen are shown for 47 task contrasts from HCP-YA. Relative scores are computed against the corresponding baseline linear regression[6] model's performance, indicating the models' performance gain or loss compared to the baseline linear model (i.e., $Relative\ Performance = ((Model\ Performance - Baseline\ Performance)\ /\ Baseline\ Performance) * 100$). Positive values indicate performance gains, and negative values indicate losses relative to the baseline. Model variants include: DeepTaskGen (proposed method); BrainSurfCNN - MSE: 50 epochs with MSE loss (i.e., first training); BrainSurfCNN - RC: Final model presented in Ngo et al., 2022[4]. Raw performance values are provided in Supplementary Figs. 6-8. DeepTaskGen achieves greater gains over the baseline than the surface-based BrainSurfCNN.

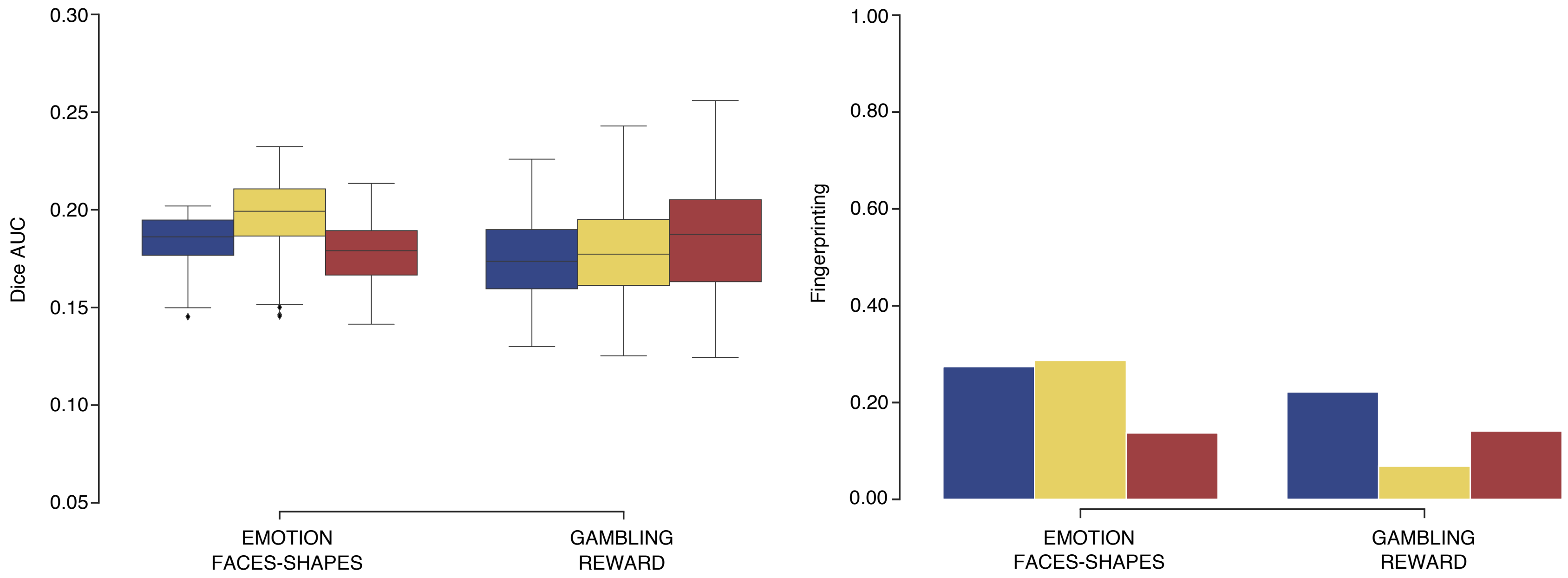

Supplementary Figure 11. Dice AUC and fingerprinting scores for fine-tuned and non-fine-tuned DeepTaskGen models, as well as the baseline linear model, for the EMOTION FACES-SHAPES and GAMBLING REWARD task contrasts from HCP-D. In boxplots, the box ranges from the first quartile to the third quartile, with a line inside indicating the median. The "whiskers" extend to the most extreme values within 1.5 times the interquartile range, which are not considered outliers. Any points outside this range are plotted individually as outliers.

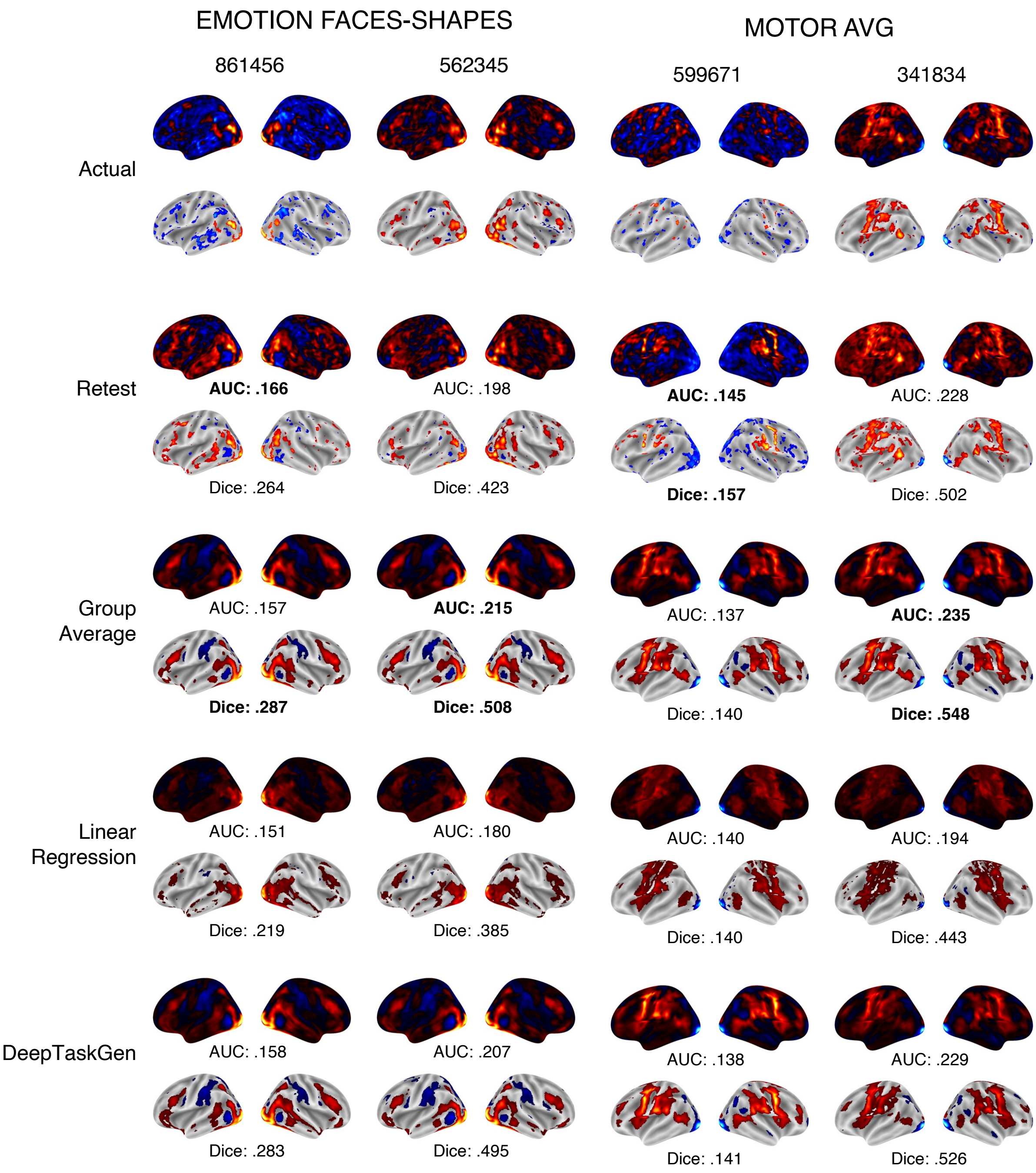

Supplementary Figure 12. Unthresholded and thresholded task activations for EMOTION FACES-SHAPES and MOTOR AVG contrasts are presented for sample atypical and typical subjects. In each task contrast map, the left column represents an atypical subject, while the right column represents a typical subject, defined by their similarity to the corresponding group average task activations. For each method, unthresholded task activations are displayed at the top, and thresholded activations (top 25% most activated voxels) are shown at the bottom. Dice AUC scores for unthresholded maps and Dice scores for thresholded maps are provided below the corresponding images.

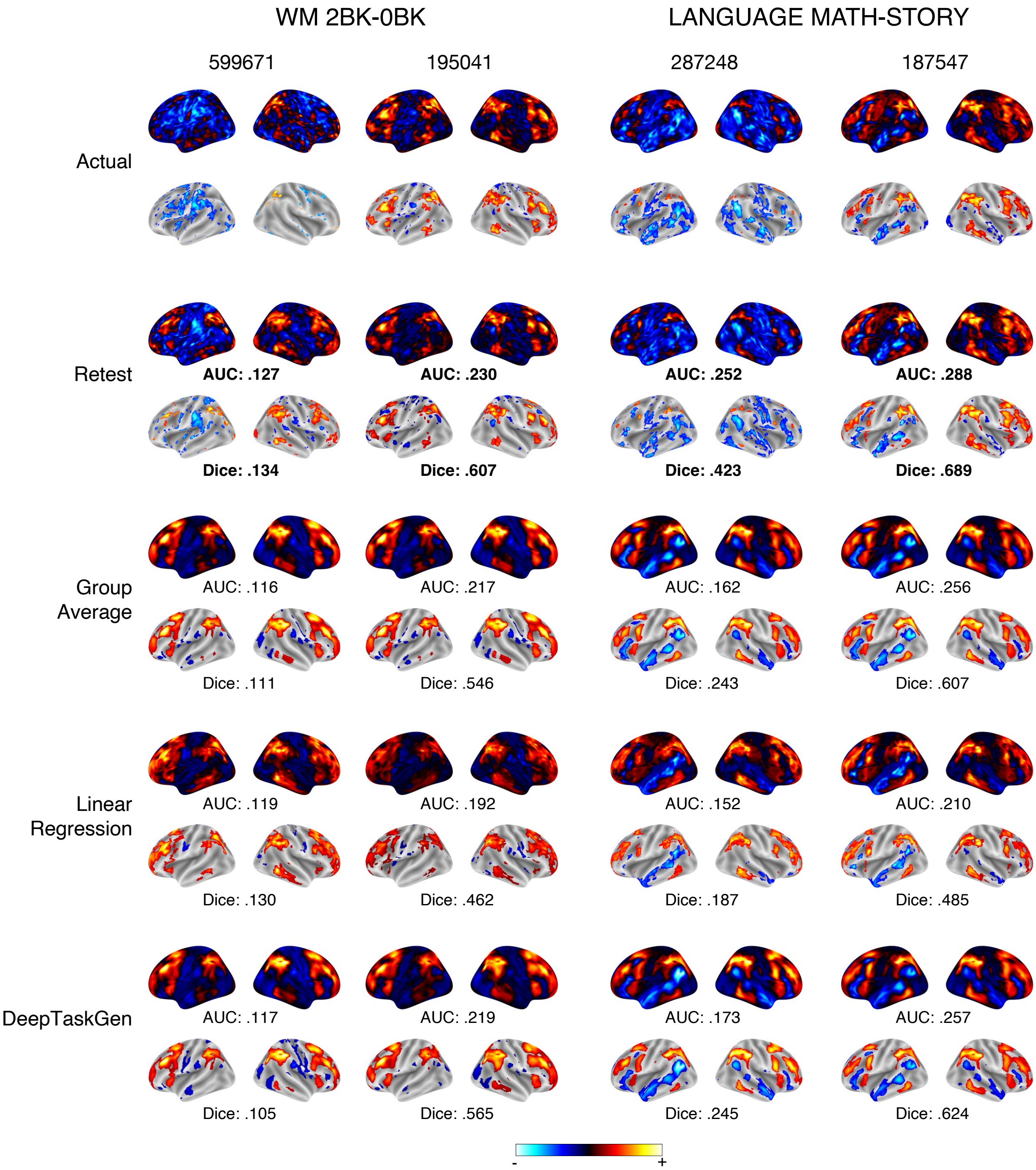

Supplementary Figure 13. Unthresholded and thresholded task activations for WM 2BK-0BK and LANGUAGE MATH-STORY contrasts are presented for sample atypical and typical subjects. In each task contrast map, the left column represents an atypical subject, while the right column represents a typical subject, defined by their similarity to the corresponding group average task activations. For each method, unthresholded task activations are displayed at the top, and thresholded activations (top 25% most activated voxels) are shown

at the bottom. Dice AUC scores for unthresholded maps and Dice scores for thresholded maps are provided below the corresponding images.

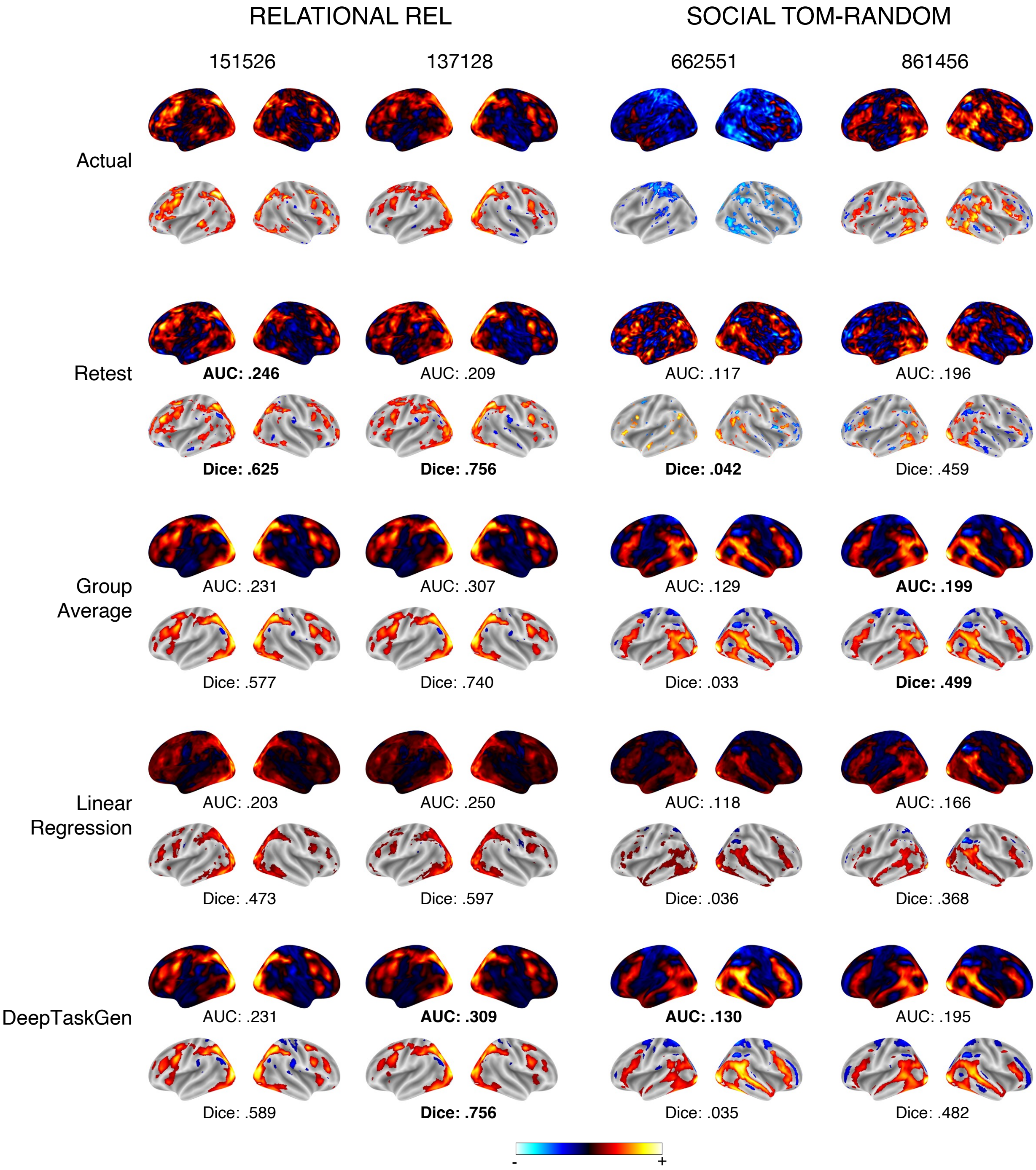

Supplementary Figure 14. Unthresholded and thresholded task activations for RELATIONAL REL and SOCIAL TOM-RANDOM contrasts are presented for sample atypical and typical subjects. In each task contrast map, the left column represents an atypical subject, while the right column represents a typical subject, defined by their similarity to the corresponding group average task activations. For each method, unthresholded task activations are

displayed at the top, and thresholded activations (top 25% most activated voxels) are shown at the bottom. Dice AUC scores for unthresholded maps and Dice scores for thresholded maps are provided below the corresponding images.

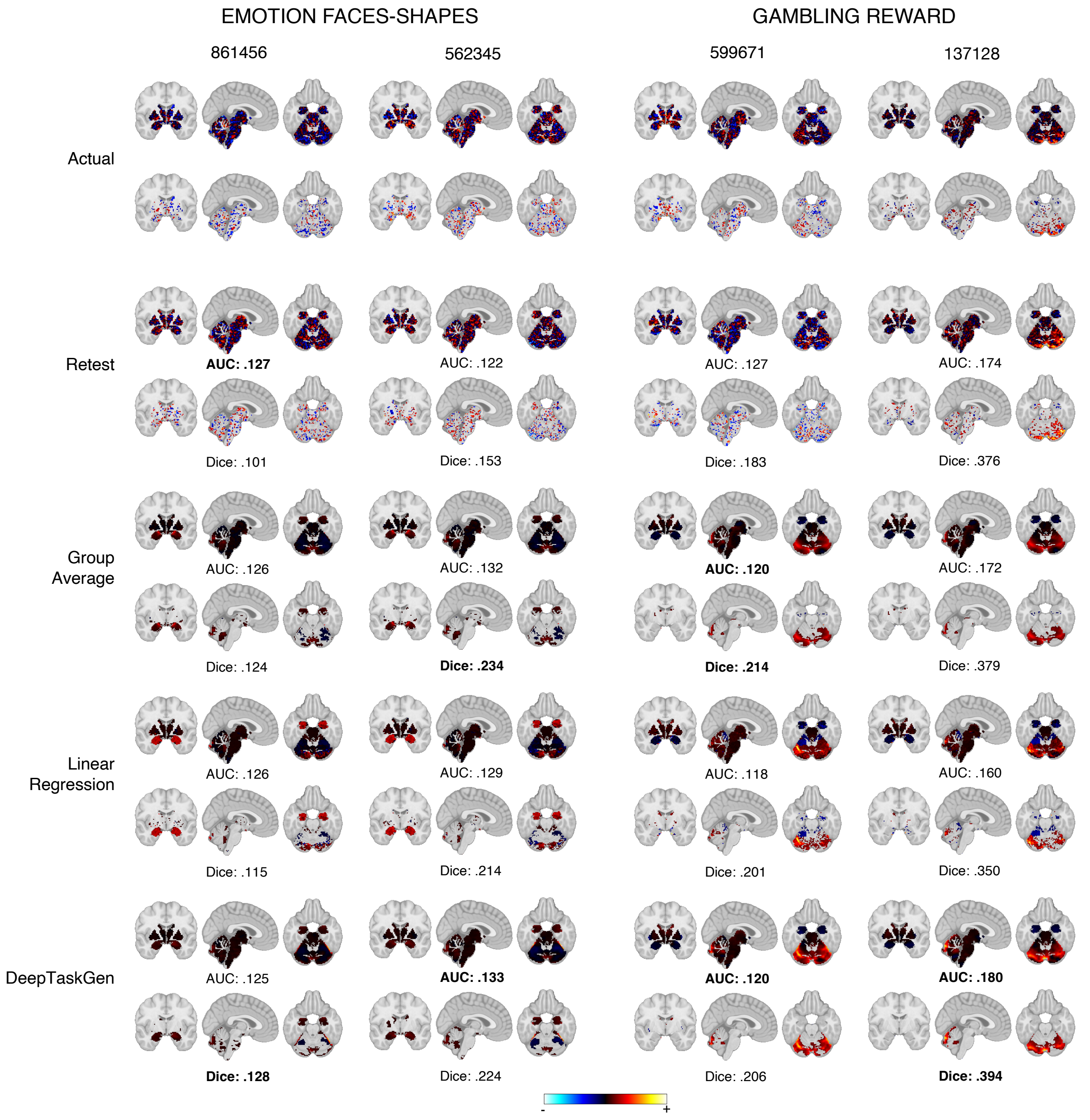

Supplementary Figure 15. Unthresholded and thresholded subcortical task activations for EMOTION FACES-SHAPES and GAMBLING REWARD contrasts are presented for sample atypical and typical subjects. In each task contrast map, the left column represents an atypical subject, while the right column represents a typical subject, defined by their similarity to the corresponding group average task activations. For each method, unthresholded task activations are displayed at the top, and thresholded activations (top 25% most activated

voxels) are shown at the bottom. Dice AUC scores for unthresholded maps and Dice scores for thresholded maps are provided below the corresponding images.

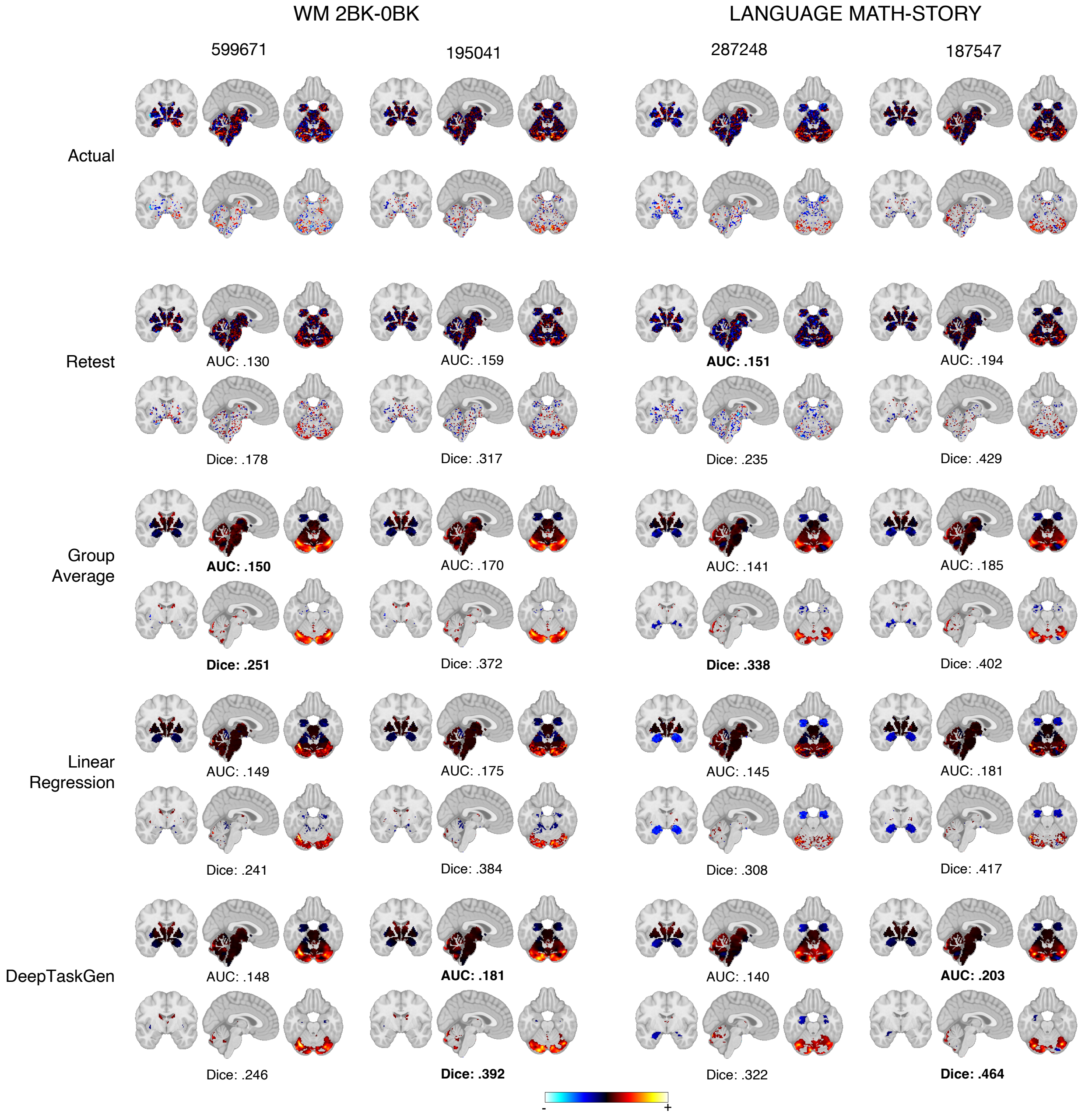

Supplementary Figure 16. Unthresholded and thresholded subcortical task activations for WM 2BK-0BK and LANGUAGE MATH-STORY contrasts are presented for sample atypical and typical subjects. In each task contrast map, the left column represents an atypical subject, while the right column represents a typical subject, defined by their similarity to the corresponding group average task activations. For each method, unthresholded task activations are displayed at the top, and thresholded activations (top 25% most activated voxels) are shown at the bottom. Dice AUC scores for unthresholded maps and Dice scores for thresholded maps are provided below the corresponding images.

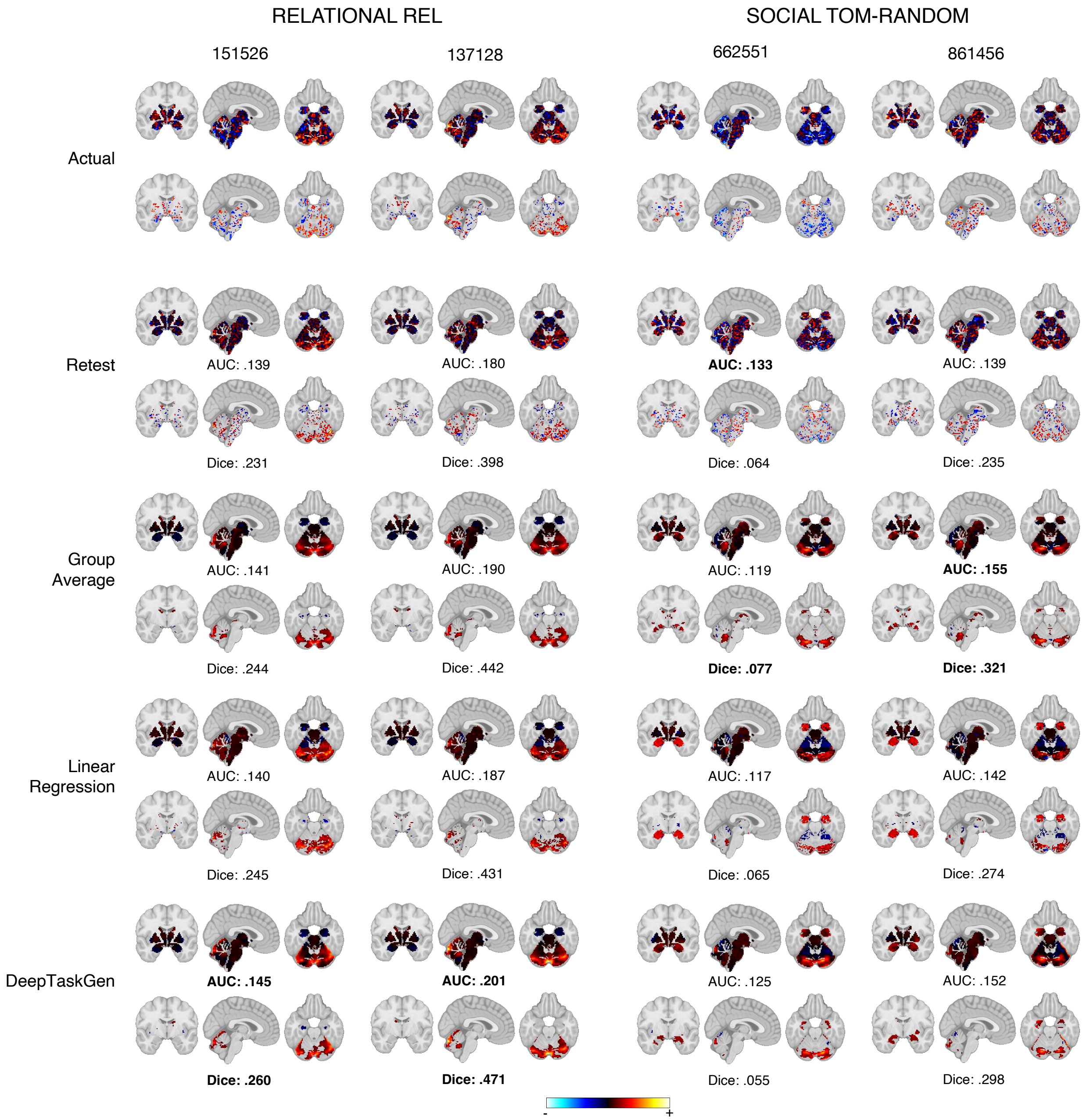

Supplementary Figure 17. Unthresholded and thresholded subcortical task activations for RELATIONAL REL and SOCIAL TOM-RANDOM contrasts are presented for sample atypical and typical subjects. In each task contrast map, the left column represents an atypical subject, while the right column represents a typical subject, defined by their similarity to the corresponding group average task activations. For each method, unthresholded task activations are displayed at the top, and thresholded activations (top 25% most activated voxels) are shown at the bottom. Dice AUC scores for unthresholded maps and Dice scores for thresholded maps are provided below the corresponding images.

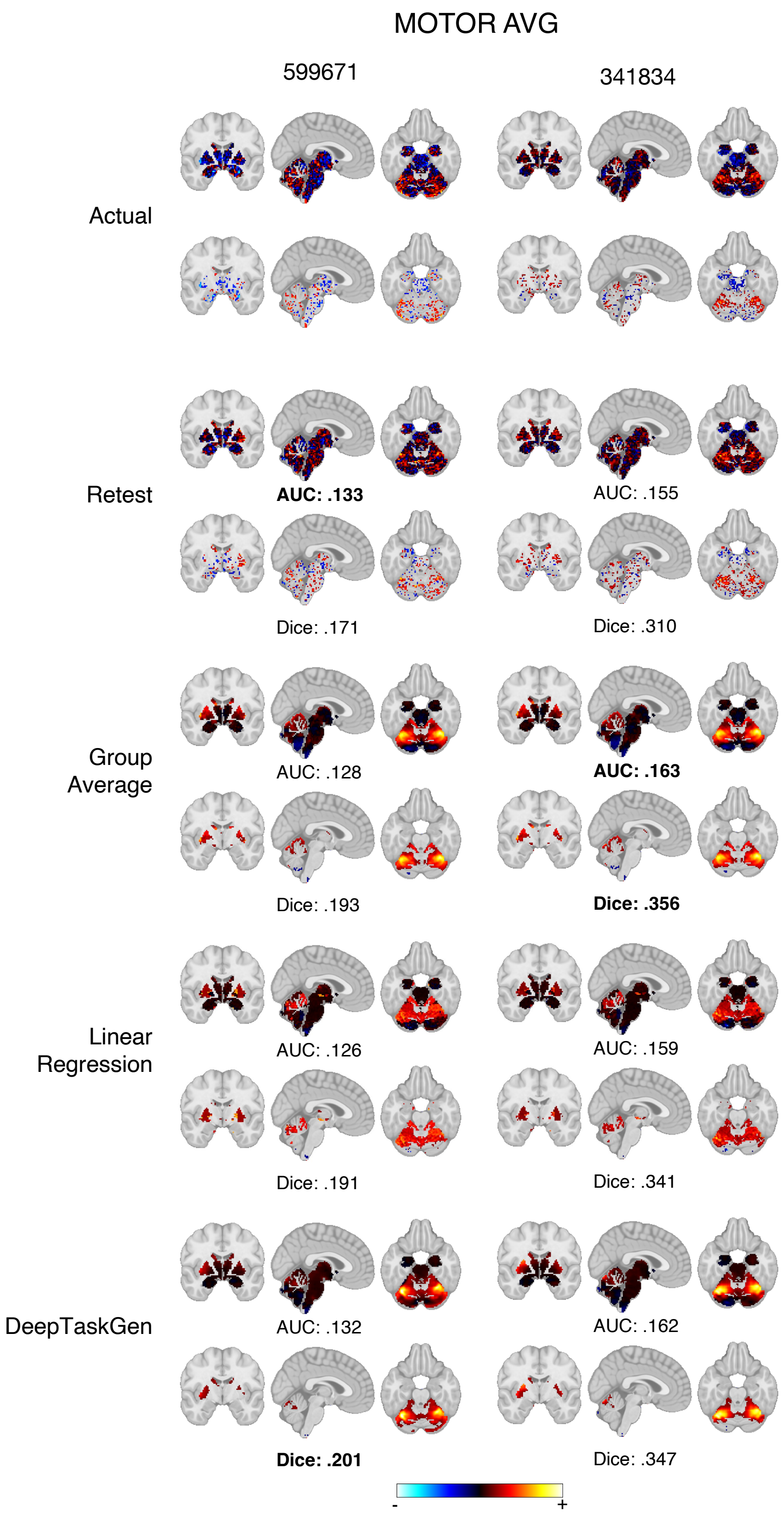

Supplementary Figure 18. Unthresholded and thresholded subcortical task activations for MOTOR AVG contrast are presented for sample atypical and typical subjects. In each task contrast map, the left column represents an atypical subject, while the right column

represents a typical subject, defined by their similarity to the corresponding group average task activations. For each method, unthresholded task activations are displayed at the top, and thresholded activations (top 25% most activated voxels) are shown at the bottom. Dice AUC scores for unthresholded maps and Dice scores for thresholded maps are provided below the corresponding images.

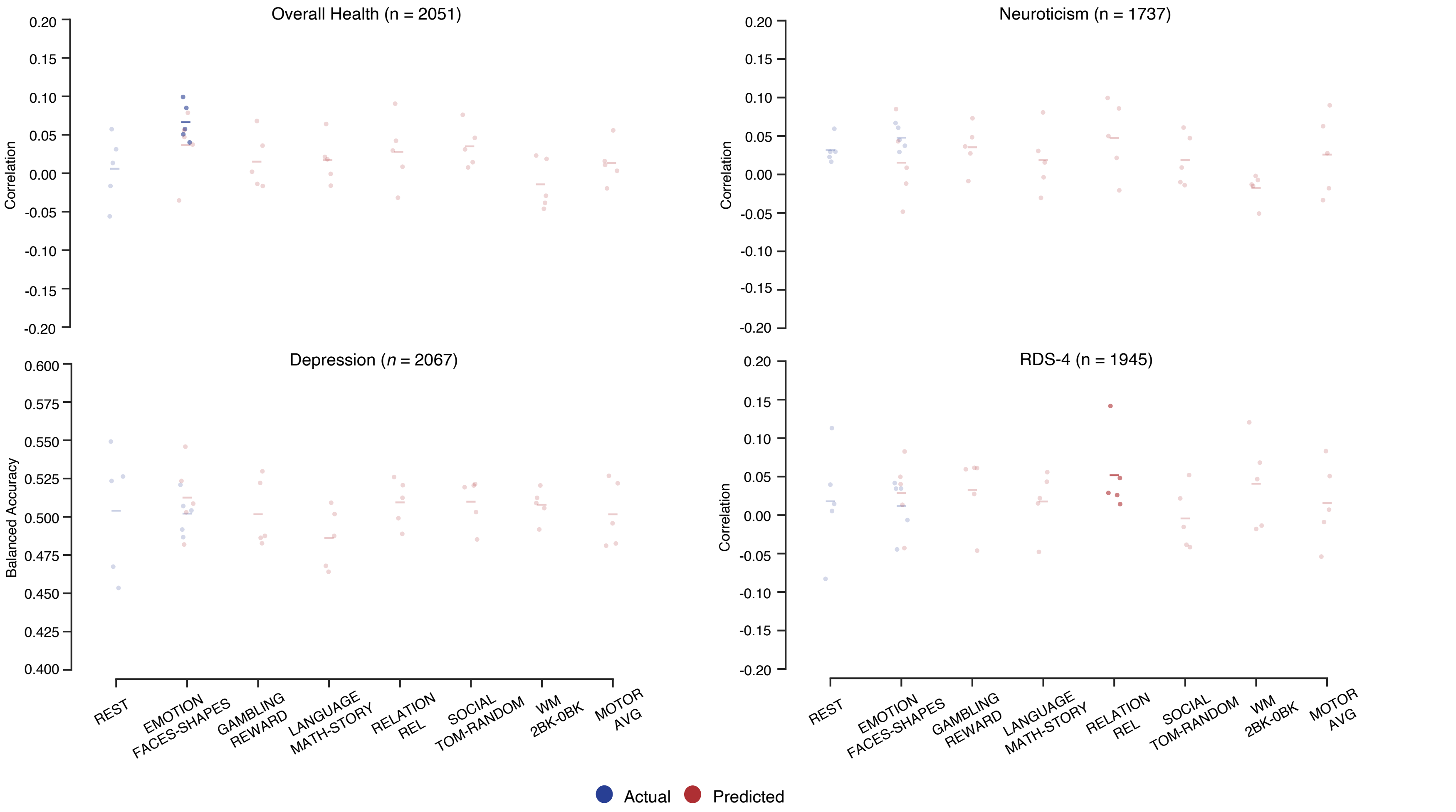

Supplementary Figure 19. Additional predictions of subjects' overall health, neuroticism, depression, and Recent Depression Symptoms-4 (RDS-4) scores using task contrast maps and resting-state connectome on UK Biobank. Actual and synthetic brain measures are depicted in blue and red colors, respectively. Significant predictions based on permutation testing are highlighted. Error bars indicate the standard deviation of prediction performance across five CV folds. Balanced accuracy was used to measure

depression classification performance, while Pearson's correlation was employed to assess other variables. Sample sizes for all analyses are indicated in each figure.

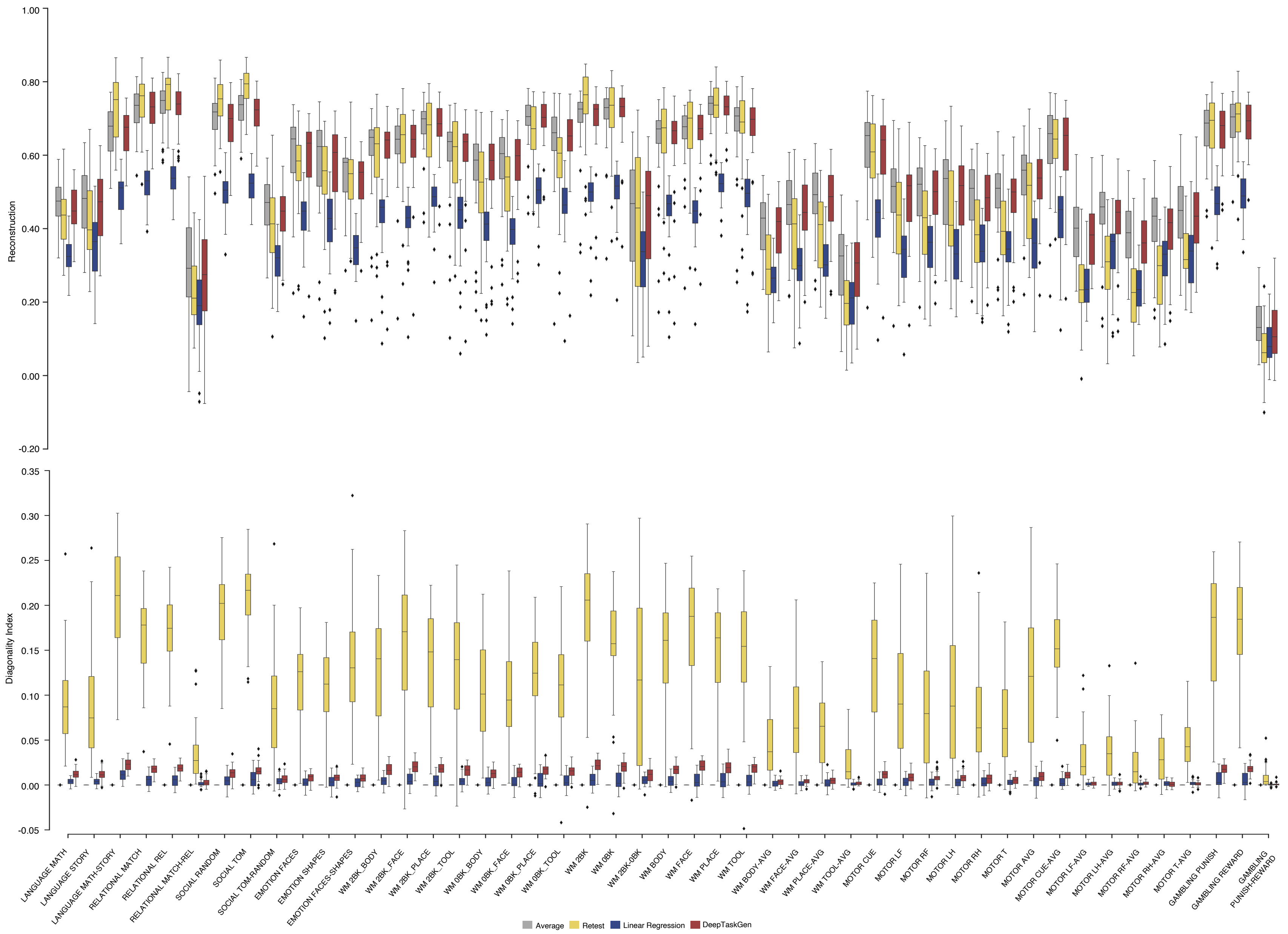
Reconstruction
1.00
0.80
0.60
0.40
0.20
0.00
-0.20
Diagonality Index
0.35
0.30
0.25
0.20
0.15
0.10
0.05
0.00
-0.05
LANGUAGE MATH
LANGUAGE STORY
LANGUAGE MATH-STORY
RELATIONAL MATCH
RELATIONAL REL
RELATIONAL MATCH-REL
SOCIAL RANDOM
SOCIAL TOM
SOCIAL TOM-RANDOM
EMOTION FACES
EMOTION SHAPES
EMOTION FACES-SHAPES
WM 2BK_BODY
WM 2BK_FACE
WM 2BK_PLACE
WM 2BK_TOOL
WM 0BK_BODY
WM 0BK_FACE
WM 0BK_PLACE
WM 0BK_TOOL
WM 2BK
WM 0BK
WM 2BK-0BK
WM BODY
WM FACE
WM PLACE
WM TOOL
WM BODY-AVG
WM FACE-AVG
WM PLACE-AVG
WM TOOL-AVG
MOTOR CUE
MOTOR LF
MOTOR RF
MOTOR LH
MOTOR RH
MOTOR T
MOTOR AVG
MOTOR CUE-AVG
MOTOR LF-AVG
MOTOR LH-AVG
MOTOR RF-AVG
MOTOR RH-AVG
MOTOR T-AVG
GAMBLING PUNISH
GAMBLING REWARD
GAMBLING PUNISH-REWARD
Average
Retest
Linear Regression
DeepTaskGen

Supplementary Figure 20. Reconstruction performance and the diagonality index of DeepTaskGen and various baselines for 47 cortical task contrasts from HCP-YA. Subcortical areas were masked out during computation of the performance metrics. In boxplots, the box ranges from the first quartile to the third quartile, with a line inside indicating the median. The "whiskers" extend to the most extreme values within 1.5 times the interquartile range, which are not considered outliers. Any points outside this range are plotted individually as outliers.

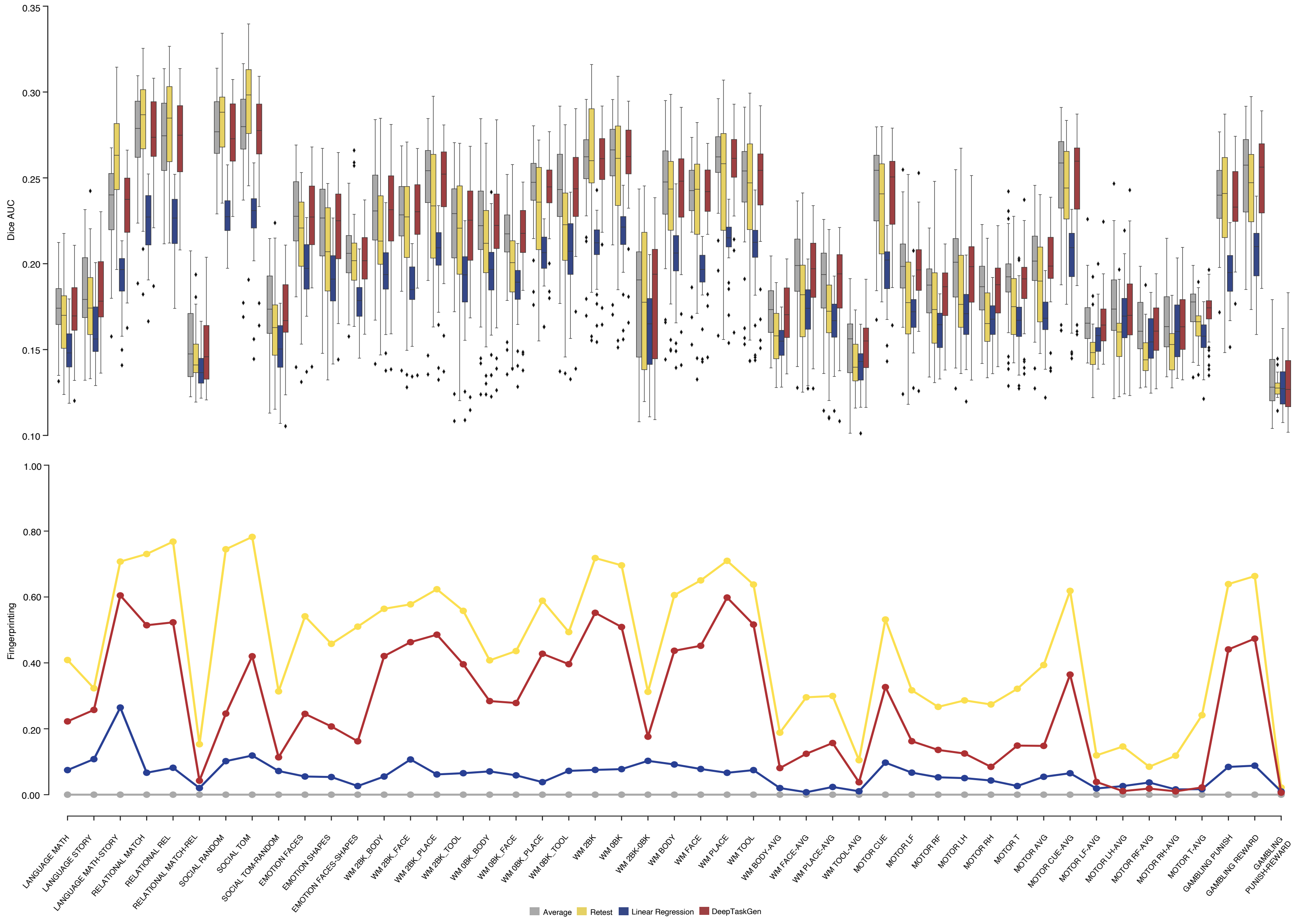

Dice AUC
0.35
0.30
0.25
0.20
0.15
0.10
Fingerprinting
1.00
0.80
0.60
0.40
0.20
0.00
LANGUAGE MATH
LANGUAGE STORY
LANGUAGE MATH-STORY
RELATIONAL MATCH
RELATIONAL REL
RELATIONAL MATCH-REL
SOCIAL RANDOM
SOCIAL TOM
SOCIAL TOM-RANDOM
EMOTION FACES
EMOTION SHAPES
EMOTION FACES-SHAPES
WM 2BK_BODY
WM 2BK_FACE
WM 2BK_PLACE
WM 2BK_TOOL
WM 0BK_BODY
WM 0BK_FACE
WM 0BK_PLACE
WM 0BK_TOOL
WM 2BK
WM 0BK
WM 2BK-0BK
WM BODY
WM FACE
WM PLACE
WM TOOL
WM BODY-AVG
WM FACE-AVG
WM PLACE-AVG
WM TOOL-AVG
MOTOR CUE
MOTOR LF
MOTOR RF
MOTOR LH
MOTOR RH
MOTOR T
MOTOR AVG
MOTOR CUE-AVG
MOTOR LF-AVG
MOTOR LH-AVG
MOTOR RF-AVG
MOTOR RH-AVG
MOTOR T-AVG
GAMBLING PUNISH
GAMBLING REWARD
GAMBLING PUNISH-REWARD
Average
Retest
Linear Regression
DeepTaskGen

Supplementary Figure 21. Dice AUC and fingerprinting score of DeepTaskGen and various baselines for 47 cortical task contrasts from HCP-YA. Subcortical areas were masked out during computation of the performance metrics. In boxplots, the box ranges from the first quartile to the third quartile, with a line inside indicating the median. The "whiskers" extend to the most extreme values within 1.5 times the interquartile range, which are not considered outliers. Any points outside this range are plotted individually as outliers.

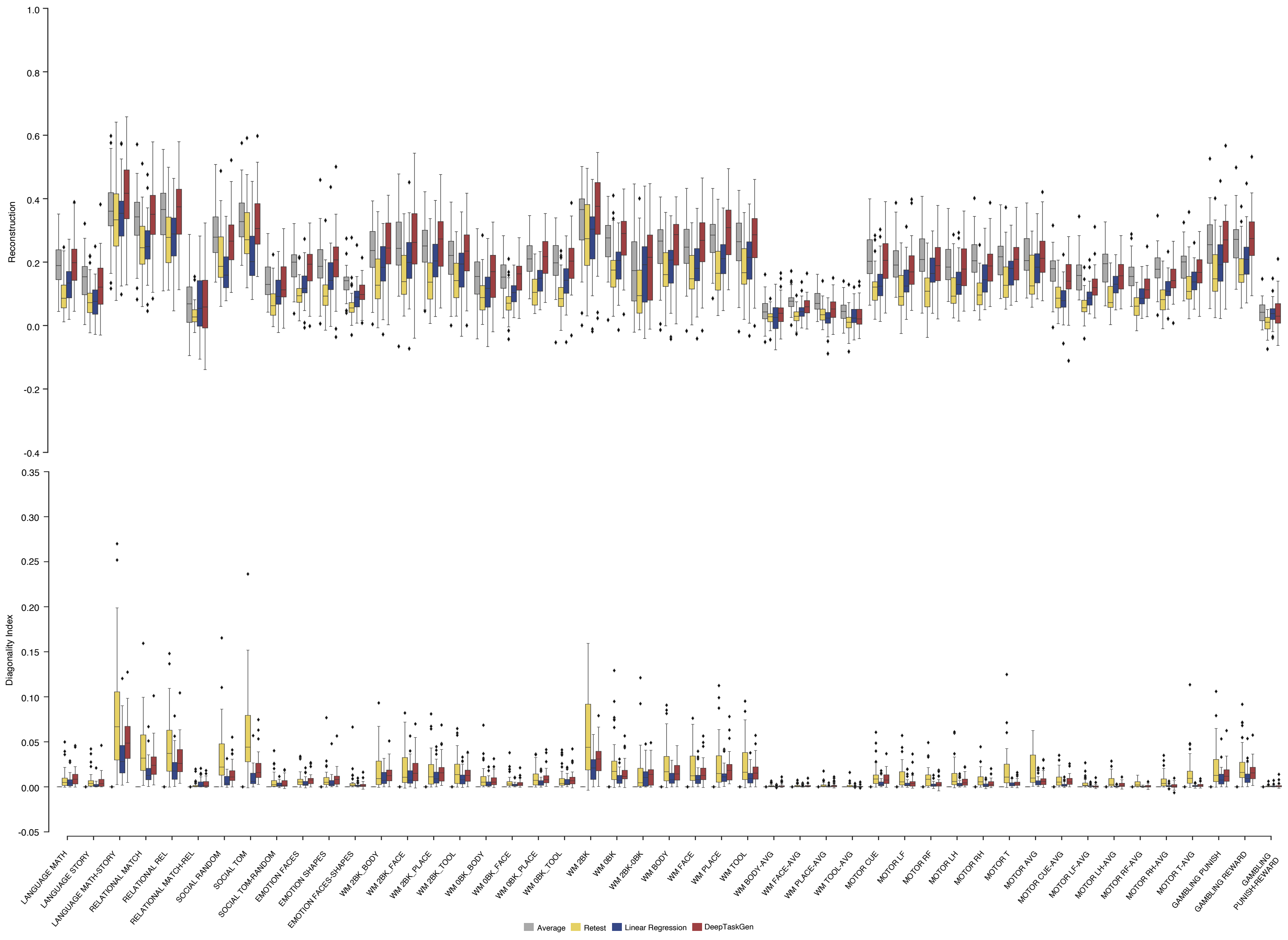

Reconstruction
1.0
0.8
0.6
0.4
0.2
0.0
-0.2
-0.4
Diagonality Index
0.35
0.30
0.25
0.20
0.15
0.10
0.05
0.00
-0.05
LANGUAGE MATH
LANGUAGE STORY
LANGUAGE MATH-STORY
RELATIONAL MATCH
RELATIONAL REL
RELATIONAL MATCH-REL
SOCIAL RANDOM
SOCIAL TOM
SOCIAL TOM-RANDOM
EMOTION FACES
EMOTION SHAPES
EMOTION FACES-SHAPES
WM 2BK_BODY
WM 2BK_FACE
WM 2BK_PLACE
WM 2BK_TOOL
WM 0BK_BODY
WM 0BK_FACE
WM 0BK_PLACE
WM 0BK_TOOL
WM 2BK
WM 0BK
WM 2BK-0BK
WM BODY
WM FACE
WM PLACE
WM TOOL
WM BODY-AVG
WM FACE-AVG
WM PLACE-AVG
WM TOOL-AVG
MOTOR CUE
MOTOR LF
MOTOR RF
MOTOR LH
MOTOR RH
MOTOR T
MOTOR AVG
MOTOR CUE-AVG
MOTOR LF-AVG
MOTOR LH-AVG
MOTOR RF-AVG
MOTOR RH-AVG
MOTOR T-AVG
GAMBLING PUNISH
GAMBLING REWARD
GAMBLING PUNISH-REWARD
Average
Retest
Linear Regression
DeepTaskGen

Supplementary Figure 22. Reconstruction performance and the diagonality index of DeepTaskGen and various baselines for 47 subcortical task contrasts from HCP-YA. Cortical areas were masked out during computation of the performance metrics. In boxplots, the box ranges from the first quartile to the third quartile, with a line inside indicating the median. The "whiskers" extend to the most extreme values within 1.5 times the interquartile range, which are not considered outliers. Any points outside this range are plotted individually as outliers.

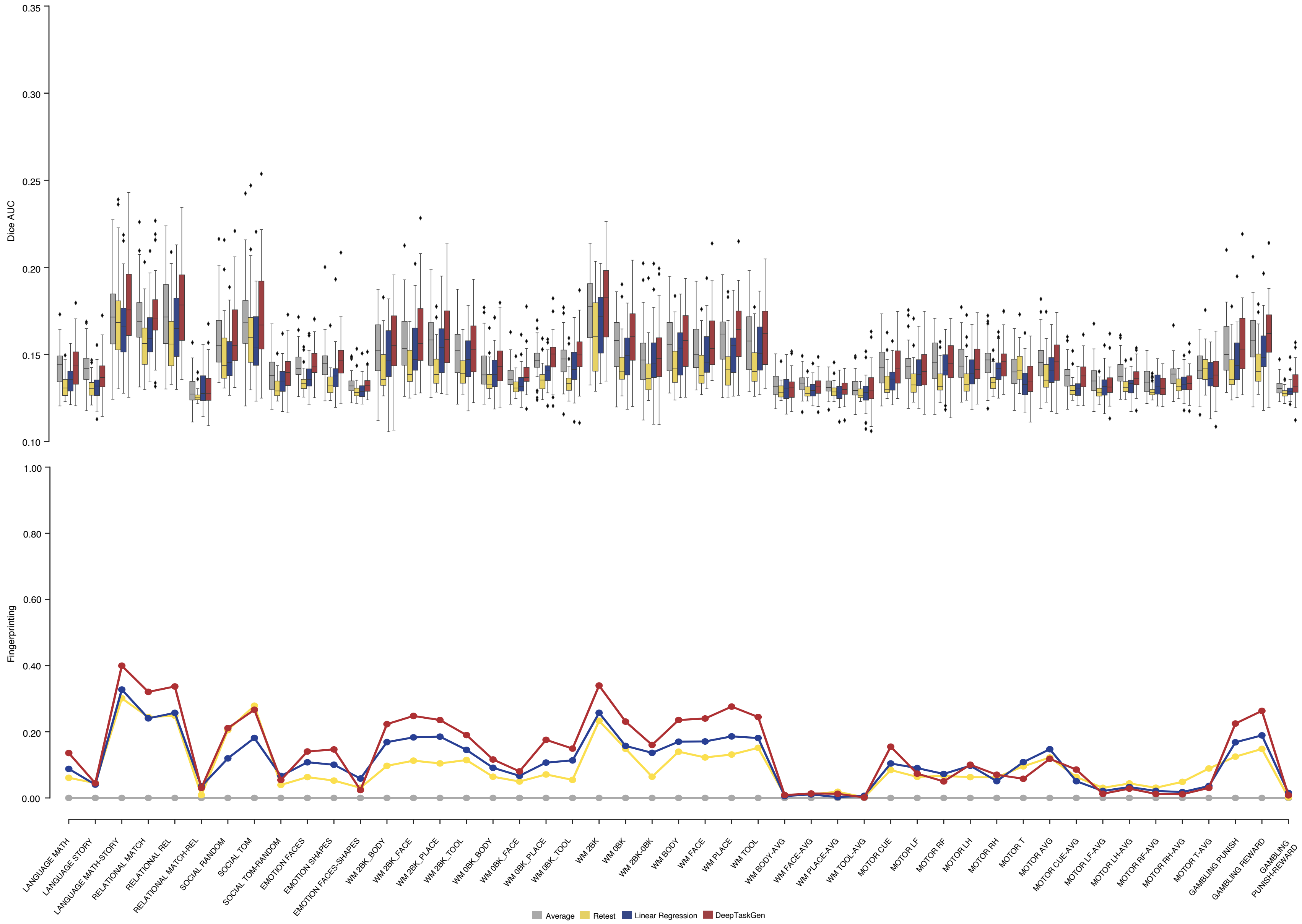

Dice AUC
Fingerprinting
LANGUAGE MATH
LANGUAGE STORY
LANGUAGE MATH-STORY
RELATIONAL MATCH
RELATIONAL REL
RELATIONAL MATCH-REL
SOCIAL RANDOM
SOCIAL TOM
SOCIAL TOM-RANDOM
EMOTION FACES
EMOTION SHAPES
EMOTION FACES-SHAPES
WM 2BK_BODY
WM 2BK_FACE
WM 2BK_PLACE
WM 2BK_TOOL
WM 0BK_BODY
WM 0BK_FACE
WM 0BK_PLACE
WM 0BK_TOOL
WM 2BK
WM 0BK
WM 2BK-0BK
WM BODY
WM FACE
WM PLACE
WM TOOL
WM BODY-AVG
WM FACE-AVG
WM PLACE-AVG
WM TOOL-AVG
MOTOR CUE
MOTOR LF
MOTOR RF
MOTOR LH
MOTOR RH
MOTOR T
MOTOR AVG
MOTOR CUE-AVG
MOTOR LF-AVG
MOTOR LH-AVG
MOTOR RF-AVG
MOTOR RH-AVG
MOTOR T-AVG
GAMBLING PUNISH
GAMBLING REWARD
GAMBLING PUNISH-REWARD
Average
Retest
Linear Regression
DeepTaskGen

Supplementary Figure 23. Dice AUC and fingerprinting score of DeepTaskGen and various baselines for 47 subcortical task contrasts from HCP-YA. Cortical areas were masked out during computation of the performance metrics. In boxplots, the box ranges from the first quartile to the third quartile, with a line inside indicating the median. The "whiskers" extend to the most extreme values within 1.5 times the interquartile range, which are not considered outliers. Any points outside this range are plotted individually as outliers.

Task Contrast	DeepTaskGen vs. Group Average			DeepTaskGen vs. Retest Scans			DeepTaskGen vs. Linear Model		
	t	*p*	*δ*	*t*	*p*	*δ*	*t*	*p*	*δ*
LANGUAGE MATH	-2.636	0.012	-0.06	2.628	0.023	0.223	17.284	0.003	0.553
LANGUAGE STORY	-1.51	0.188	-0.03	3.783	0.003	0.295	15.528	0.003	0.502
LANGUAGE MATH-STORY	-0.472	0.679	-0.005	-6.854	0.003	-0.41	25.105	0.003	0.761
RELATIONAL MATCH	-1.13	0.318	-0.015	-0.073	0.901	-0.031	32.03	0.003	0.804
RELATIONAL REL	-1.17	0.318	-0.015	-1.347	0.206	-0.09	29.418	0.003	0.761
RELATIONAL MATCH-REL	-4.431	0.006	-0.07	2.193	0.069	0.114	5.657	0.003	0.233
SOCIAL RANDOM	-5.097	0.003	-0.072	-1.401	0.185	-0.093	30.753	0.003	0.888
SOCIAL TOM	-3.996	0.003	-0.062	-3.6	0.006	-0.26	29.973	0.003	0.871
SOCIAL TOM-RANDOM	-5.751	0.003	-0.07	2.008	0.069	0.21	13.003	0.003	0.479
EMOTION FACES	-3.666	0.006	-0.041	6.123	0.003	0.327	23.305	0.003	0.628
EMOTION SHAPES	-4.593	0.003	-0.049	7.534	0.003	0.339	19.015	0.003	0.546
EMOTION FACES-SHAPES	-7.912	0.003	-0.157	0.254	0.853	0.116	20.776	0.003	0.663
WM 2BK_BODY	-1.342	0.251	-0.019	3.549	0.003	0.264	17.632	0.003	0.642
WM 2BK_FACE	-0.287	0.822	-0.009	2.268	0.03	0.147	23.297	0.003	0.819
WM 2BK_PLACE	-0.369	0.772	-0.01	3.724	0.003	0.274	22.059	0.003	0.745
WM 2BK_TOOL	-2.047	0.087	-0.016	1.817	0.104	0.114	16.789	0.003	0.549
WM 0BK_BODY	-0.7	0.531	-0.007	4.35	0.003	0.265	15.486	0.003	0.491
WM 0BK_FACE	0.038	0.967	-0.015	6.005	0.003	0.485	17.403	0.003	0.711
WM 0BK_PLACE	-2.235	0.043	-0.043	4.034	0.003	0.31	24.358	0.003	0.813
WM 0BK_TOOL	-0.688	0.5	-0.002	6.069	0.003	0.408	18.755	0.003	0.612
WM 2BK	-0.877	0.444	-0.022	-0.192	0.859	-0.035	24.572	0.003	0.841
WM 0BK	-1.123	0.309	-0.027	2.535	0.021	0.165	23.702	0.003	0.82
WM 2BK-0BK	-0.499	0.676	-0.001	1.265	0.286	0.078	8.584	0.003	0.294

WM BODY	-1.434	0.214	-0.012	2.61	0.021	0.16	19.199	0.003	0.684
WM FACE	0.129	0.901	-0.002	2.454	0.03	0.135	24.23	0.003	0.846
WM PLACE	-0.728	0.503	-0.037	2.681	0.023	0.191	27.206	0.003	0.854
WM TOOL	-0.891	0.444	-0.005	2.262	0.038	0.126	21.363	0.003	0.715
WM BODY-AVG	-3.975	0.006	-0.057	6.991	0.003	0.386	12.542	0.003	0.486
WM FACE-AVG	-5.979	0.003	-0.062	5.217	0.003	0.295	15.62	0.003	0.46
WM PLACE-AVG	-3.093	0.009	-0.043	5.854	0.003	0.454	16.196	0.003	0.554
WM TOOL-AVG	-2.282	0.04	-0.049	3.271	0.003	0.35	8.526	0.003	0.35
MOTOR CUE	-5.969	0.003	-0.111	4.52	0.003	0.319	27.407	0.003	0.744
MOTOR LF	-3.765	0.006	-0.059	5.372	0.003	0.402	14.637	0.003	0.645
MOTOR RF	-1.999	0.05	-0.055	2.315	0.047	0.249	15.864	0.003	0.603
MOTOR LH	-3.698	0.006	-0.055	4.322	0.003	0.349	17.082	0.003	0.591
MOTOR RH	-1.87	0.086	-0.028	7.107	0.003	0.482	14.194	0.003	0.515
MOTOR T	-3.466	0.003	-0.045	4.267	0.003	0.261	18.168	0.003	0.495
MOTOR AVG	-1.414	0.203	-0.02	3.403	0.006	0.275	17.813	0.003	0.734
MOTOR CUE-AVG	-4.995	0.003	-0.051	3.407	0.006	0.248	23.216	0.003	0.67
MOTOR LF-AVG	-5.065	0.003	-0.081	6.604	0.003	0.596	7.385	0.003	0.236
MOTOR LH-AVG	-8.699	0.003	-0.093	4.659	0.003	0.34	4.297	0.006	0.09
MOTOR RF-AVG	-8.18	0.003	-0.11	5.968	0.003	0.436	7.07	0.003	0.145
MOTOR RH-AVG	-4.931	0.003	-0.07	6.586	0.003	0.423	5.286	0.003	0.101
MOTOR T-AVG	-6.943	0.003	-0.157	2.397	0.023	0.249	11.082	0.003	0.566
GAMBLING PUNISH	-2.211	0.033	-0.034	1.78	0.118	0.086	28.519	0.003	0.862
GAMBLING REWARD	-2.439	0.025	-0.04	2.025	0.057	0.211	30.51	0.003	0.771
GAMBLING PUNISH-REWARD	-3.412	0.003	-0.072	2.628	0.023	0.223	17.284	0.003	0.553

Supplementary Table 1: Paired t-tests with permutation testing ($P = 1000$) were performed to compare DeepTaskGen's Dice AUC score with various baseline methods, including group-average task contrast maps, retest scans, and the linear model on HCP-YA. The sample size for this comparison was 39. *p* values are FDR corrected for 3 pairwise comparisons across 47 task contrasts. Cliff's Delta (δ) was used to measure effect size.

Task Contrast	Group Average	Retest Scans	Linear Model	DeepTaskGen
LANGUAGE MATH	0	**0.337**	0.111	0.256
LANGUAGE STORY	0	**0.281**	0.119	0.243
LANGUAGE MATH-STORY	0	**0.671**	0.341	0.601
RELATIONAL MATCH	0	**0.696**	0.078	0.518
RELATIONAL REL	0	**0.735**	0.120	0.547
RELATIONAL MATCH-REL	0	**0.114**	0.032	0.051
SOCIAL RANDOM	0	**0.711**	0.089	0.310
SOCIAL TOM	0	**0.751**	0.131	0.411
SOCIAL TOM-RANDOM	0	**0.260**	0.080	0.135
EMOTION FACES	0	**0.478**	0.053	0.247
EMOTION SHAPES	0	**0.401**	0.051	0.196
EMOTION FACES-SHAPES	0	**0.460**	0.025	0.153
WM 2BK_BODY	0	**0.489**	0.064	0.416
WM 2BK_FACE	0	**0.533**	0.103	0.474
WM 2BK_PLACE	0	**0.562**	0.083	0.516
WM 2BK_TOOL	0	**0.498**	0.062	0.390
WM 0BK_BODY	0	**0.357**	0.077	0.269
WM 0BK_FACE	0	**0.392**	0.056	0.277
WM 0BK_PLACE	0	**0.544**	0.037	0.393
WM 0BK_TOOL	0	**0.449**	0.081	0.409
WM 2BK	0	**0.685**	0.098	0.555
WM 0BK	0	**0.641**	0.076	0.494
WM 2BK-0BK	0	**0.279**	0.149	0.227
WM BODY	0	**0.565**	0.100	0.436
WM FACE	0	**0.591**	0.097	0.484
WM PLACE	0	**0.635**	0.065	0.562
WM TOOL	0	**0.597**	0.096	0.498

WM BODY-AVG	0	**0.159**	0.018	0.082
WM FACE-AVG	0	**0.263**	0.007	0.104
WM PLACE-AVG	0	**0.248**	0.021	0.155
WM TOOL-AVG	0	**0.081**	0.009	0.040
MOTOR CUE	0	**0.481**	0.099	0.382
MOTOR LF	0	**0.272**	0.078	0.175
MOTOR RF	0	**0.220**	0.041	0.104
MOTOR LH	0	**0.258**	0.071	0.175
MOTOR RH	0	**0.254**	0.048	0.079
MOTOR T	0	**0.284**	0.033	0.149
MOTOR AVG	0	**0.347**	0.060	0.165
MOTOR CUE-AVG	0	**0.579**	0.066	0.341
MOTOR LF-AVG	0	**0.091**	0.006	0.035
MOTOR LH-AVG	0	**0.134**	0.032	0.040
MOTOR RF-AVG	0	**0.083**	0.039	0.017
MOTOR RH-AVG	0	**0.104**	0.015	0.028
MOTOR T-AVG	0	**0.219**	0.023	0.020
GAMBLING PUNISH	0	**0.565**	0.117	0.458
GAMBLING REWARD	0	**0.624**	0.135	0.475
GAMBLING PUNISH-REWARD	0	**0.017**	0.011	0.011

Supplementary Table 2: The fingerprinting scores outlined in the main manuscript. The table presents the results obtained from analyses performed on the HCP-YA dataset. The highest fingerprinting score for each task contrast is highlighted, indicating the method that achieved the best performance in differentiating between individuals based on their task contrast maps.

Task Contrast	DeepTaskGen vs. Group Average			DeepTaskGen vs. Retest Scans			DeepTaskGen vs. Linear Model		
	t	*p*	*δ*	*t*	*p*	*δ*	*t*	*p*	*δ*
LANGUAGE MATH	-4.996	-0.119	0.003	4.227	0.339	0.003	27.981	0.85	0.003
LANGUAGE STORY	-4.589	-0.099	0.003	4.607	0.335	0.003	29.489	0.553	0.003
LANGUAGE MATH-STORY	-1.367	-0.012	0.179	-6.111	-0.395	0.003	45.423	0.859	0.003
RELATIONAL MATCH	-2.058	-0.024	0.072	-2.125	-0.126	0.05	50.555	0.955	0.003
RELATIONAL REL	-1.744	-0.023	0.09	-3.144	-0.195	0.013	54.825	0.958	0.003
RELATIONAL MATCH-REL	-11.443	-0.111	0.003	3.238	0.285	0.005	11.626	0.302	0.003
SOCIAL RANDOM	-7.177	-0.111	0.003	-5.168	-0.324	0.003	41.246	0.941	0.003
SOCIAL TOM	-5.418	-0.101	0.003	-8.297	-0.499	0.003	48.664	0.989	0.003
SOCIAL TOM-RANDOM	-9.617	-0.149	0.003	4.289	0.302	0.003	24.233	0.788	0.003
EMOTION FACES	-8.689	-0.128	0.003	6.237	0.332	0.003	28.483	0.841	0.003
EMOTION SHAPES	-7.318	-0.108	0.003	6.256	0.298	0.003	26.222	0.726	0.003
EMOTION FACES-SHAPES	-9.908	-0.147	0.003	1.009	0.122	0.297	31.08	0.905	0.003
WM 2BK_BODY	-1.987	-0.045	0.062	3.174	0.204	0.003	27.361	0.836	0.003
WM 2BK_FACE	-0.15	0.005	0.919	1.331	0.06	0.235	32.019	0.913	0.003
WM 2BK_PLACE	-1.058	-0.01	0.341	4.454	0.224	0.003	38.255	0.904	0.003
WM 2BK_TOOL	-1.413	-0.015	0.192	1.917	0.124	0.09	25.06	0.761	0.003
WM 0BK_BODY	-2.186	-0.014	0.046	5.316	0.329	0.003	23.219	0.758	0.003
WM 0BK_FACE	-2.568	-0.039	0.023	7.866	0.461	0.003	27.307	0.849	0.003
WM 0BK_PLACE	-0.972	-0.024	0.35	5.448	0.378	0.003	46.263	0.911	0.003
WM 0BK_TOOL	-1.339	-0.01	0.199	7.088	0.486	0.003	32.31	0.888	0.003
WM 2BK	-0.507	-0.014	0.643	-2.218	-0.302	0.052	35.731	0.892	0.003
WM 0BK	-0.82	-0.015	0.432	1.267	0.051	0.235	37.632	0.888	0.003
WM 2BK-0BK	-0.456	-0.011	0.663	1.745	0.061	0.108	14.1	0.448	0.003
WM BODY	-2.044	-0.034	0.048	1.138	0.053	0.342	26.863	0.804	0.003

WM FACE	-1.006	-0.01	0.344	0.564	-0.069	0.613	32.556	0.9	0.003
WM PLACE	-0.904	-0.014	0.39	2.166	0.093	0.07	52.646	0.993	0.003
WM TOOL	-0.985	-0.018	0.337	1.432	0.089	0.223	32.486	0.854	0.003
WM BODY-AVG	-6.535	-0.098	0.003	11.004	0.621	0.003	19.991	0.775	0.003
WM FACE-AVG	-8.533	-0.083	0.003	6.66	0.377	0.003	21.899	0.717	0.003
WM PLACE-AVG	-7.555	-0.066	0.003	8.988	0.528	0.003	24.026	0.779	0.003
WM TOOL-AVG	-7.475	-0.089	0.003	7.225	0.584	0.003	18.31	0.477	0.003
MOTOR CUE	-6.986	-0.091	0.003	3.557	0.273	0.003	35.202	0.819	0.003
MOTOR LF	-5.479	-0.068	0.003	4.813	0.377	0.003	26.598	0.767	0.003
MOTOR RF	-7.021	-0.111	0.003	5.078	0.379	0.003	22.516	0.717	0.003
MOTOR LH	-6.661	-0.077	0.003	4.974	0.302	0.003	24.779	0.702	0.003
MOTOR RH	-6.013	-0.086	0.003	5.429	0.433	0.003	22.357	0.686	0.003
MOTOR T	-6.994	-0.094	0.003	8.026	0.479	0.003	24.727	0.715	0.003
MOTOR AVG	-4.245	-0.07	0.003	2.982	0.227	0.008	27.722	0.788	0.003
MOTOR CUE-AVG	-7.689	-0.08	0.003	0.924	0.108	0.395	41.548	0.807	0.003
MOTOR LF-AVG	-11.464	-0.139	0.003	10.545	0.763	0.003	19.938	0.763	0.003
MOTOR LH-AVG	-12.7	-0.156	0.003	10.4	0.669	0.003	20.203	0.545	0.003
MOTOR RF-AVG	-12.943	-0.143	0.003	13.923	0.749	0.003	18.696	0.732	0.003
MOTOR RH-AVG	-12.459	-0.145	0.003	11.155	0.678	0.003	20.569	0.511	0.003
MOTOR T-AVG	-14.479	-0.108	0.003	8.646	0.563	0.003	22.381	0.55	0.003
GAMBLING PUNISH	-2.401	-0.047	0.016	0.756	0.016	0.508	43.316	0.909	0.003
GAMBLING REWARD	-1.888	-0.039	0.064	0.44	0.005	0.704	45.671	0.954	0.003
GAMBLING PUNISH-REWARD	-6.999	-0.265	0.003	4.227	0.339	0.003	27.981	0.85	0.003

Supplementary Table 3: Paired t-tests with permutation testing $(P = 1000)$ were performed to compare DeepTaskGen's reconstruction performance with various baseline methods, including group-average task contrast maps, retest scans, and the linear model on HCP-YA. The sample size for this comparison was 39. p values are FDR corrected for 3 pairwise comparisons across 47 task contrasts. Cliff's Delta (δ) was used to measure effect size.

Task Contrast	DeepTaskGen vs. Group Average			DeepTaskGen vs. Retest Scans			DeepTaskGen vs. Linear Model		
	t	*p*	*δ*	*t*	*p*	*δ*	*t*	*p*	*δ*
LANGUAGE MATH	11.608	0.002	0.949	-9.596	0.002	-0.97	8.008	0.002	0.704
LANGUAGE STORY	10.268	0.002	0.846	-7.628	0.002	-0.886	7.799	0.002	0.661
LANGUAGE MATH-STORY	20.287	0.002	1.00	-16.298	0.002	-1.00	11.147	0.002	0.72
RELATIONAL MATCH	17.639	0.002	1.00	-18.616	0.002	-0.997	8.974	0.002	0.805
RELATIONAL REL	19.094	0.002	1.00	-20.284	0.002	-1.00	9.496	0.002	0.808
RELATIONAL MATCH-REL	4.593	0.002	0.487	-5.755	0.002	-0.78	2.479	0.032	0.204
SOCIAL RANDOM	10.275	0.002	0.897	-22.657	0.002	-1.00	4.279	0.002	0.508
SOCIAL TOM	12.872	0.002	0.949	-28.626	0.002	-1.00	5.368	0.002	0.577
SOCIAL TOM-RANDOM	7.303	0.002	0.846	-7.39	0.002	-0.832	2.817	0.015	0.258
EMOTION FACES	10.316	0.002	0.949	-13.586	0.002	-0.938	4.741	0.002	0.49
EMOTION SHAPES	7.361	0.002	0.744	-11.191	0.002	-0.844	3.356	0.004	0.37
EMOTION FACES-SHAPES	9.82	0.002	0.897	-10.697	0.002	-0.997	4.426	0.002	0.482
WM 2BK_BODY	13.248	0.002	0.949	-10.754	0.002	-0.913	8.622	0.002	0.744
WM 2BK_FACE	15.787	0.002	1.00	-11.151	0.002	-0.838	10.323	0.002	0.808
WM 2BK_PLACE	14.629	0.002	0.949	-11.014	0.002	-0.95	8.446	0.002	0.771
WM 2BK_TOOL	13.03	0.002	0.949	-10.594	0.002	-0.871	8.22	0.002	0.695
WM 0BK_BODY	10.845	0.002	0.846	-9.003	0.002	-0.807	6.228	0.002	0.637
WM 0BK_FACE	13.443	0.002	0.949	-9.776	0.002	-0.854	6.838	0.002	0.708
WM 0BK_PLACE	14.396	0.002	0.949	-10.52	0.002	-0.811	7.519	0.002	0.663
WM 0BK_TOOL	12.971	0.002	0.949	-9.386	0.002	-0.851	6.43	0.002	0.641
WM 2BK	16.803	0.002	1.00	-14.447	0.002	-0.949	9.949	0.002	0.817
WM 0BK	14.339	0.002	0.949	-13.561	0.002	-0.913	7.533	0.002	0.725
WM 2BK-0BK	8.813	0.002	0.897	-6.878	0.002	-0.608	5.558	0.002	0.394
WM BODY	13.467	0.002	0.949	-12.345	0.002	-0.876	8.052	0.002	0.732

WM FACE	16.113	0.002	0.949	-12.982	0.002	-0.909	9.278	0.002	0.809
WM PLACE	15.775	0.002	1.00	-13.284	0.002	-0.943	8.567	0.002	0.748
WM TOOL	14.828	0.002	1.00	-12.586	0.002	-0.941	7.92	0.002	0.725
WM BODY-AVG	5.448	0.002	0.59	-7.098	0.002	-0.786	2.714	0.015	0.293
WM FACE-AVG	7.433	0.002	0.641	-7.921	0.002	-0.855	3.324	0.004	0.408
WM PLACE-AVG	6.569	0.002	0.795	-8.481	0.002	-0.804	2.414	0.036	0.337
WM TOOL-AVG	5.09	0.002	0.641	-6.142	0.002	-0.663	2.075	0.058	0.248
MOTOR CUE	12.452	0.002	0.949	-11.308	0.002	-0.946	7.891	0.002	0.708
MOTOR LF	9.191	0.002	0.949	-7.807	0.002	-0.749	5.788	0.002	0.488
MOTOR RF	8.827	0.002	0.897	-7.102	0.002	-0.804	4.613	0.002	0.487
MOTOR LH	9.154	0.002	0.897	-7.259	0.002	-0.779	5.161	0.002	0.486
MOTOR RH	6.78	0.002	0.795	-6.967	0.002	-0.801	3.621	0.002	0.324
MOTOR T	7.652	0.002	0.846	-8.14	0.002	-0.942	3.082	0.002	0.282
MOTOR AVG	9.475	0.002	1.00	-8.579	0.002	-0.905	4.917	0.002	0.452
MOTOR CUE-AVG	12.945	0.002	1.00	-16.503	0.002	-1.00	7.409	0.002	0.645
MOTOR LF-AVG	4.165	0.002	0.385	-5.623	0.002	-0.74	1.106	0.243	0.106
MOTOR LH-AVG	3.062	0.007	0.385	-6.383	0.002	-0.695	0.88	0.363	0.09
MOTOR RF-AVG	5.46	0.002	0.59	-4.714	0.002	-0.54	1.896	0.068	0.25
MOTOR RH-AVG	2.514	0.019	0.282	-7.143	0.002	-0.694	-0.594	0.557	-0.061
MOTOR T-AVG	4.392	0.004	0.487	-9.104	0.002	-0.992	-0.429	0.653	-0.043
GAMBLING PUNISH	16.275	0.002	1.00	-13.436	0.002	-0.966	8.211	0.002	0.695
GAMBLING REWARD	18.129	0.002	1.00	-15.527	0.002	-0.996	7.722	0.002	0.713
GAMBLING PUNISH-REWARD	2.676	0.002	0.333	-9.596	0.002	-0.97	8.008	0.002	0.704

Supplementary Table 4: Paired t-tests with permutation testing $(P = 1000)$ were performed to compare DeepTaskGen's Diagonality Index scores with various baseline methods, including group-average task contrast maps, retest scans, and the linear model on HCP-YA. The sample size for this comparison was 39. p values are FDR corrected for 3 pairwise comparisons across 47 task contrasts. Cliff's Delta (δ) was used to measure effect size.

Task Contrast	Group Average Volume vs. Group Average Surface			Retest Volume vs. Retest Surface		
	t	*p*	*δ*	*t*	*p*	*δ*
LANGUAGE MATH	-1.436	0.174	-0.061	-3.266	0.003	-0.078
LANGUAGE STORY	-0.314	0.794	0.002	-1.675	0.111	-0.040
LANGUAGE MATH-STORY	-4.705	0.003	-0.077	-2.748	0.012	-0.034
RELATIONAL MATCH	0.268	0.786	0.022	-1.551	0.181	-0.010
RELATIONAL REL	-0.755	0.494	0.009	-1.125	0.317	-0.012
RELATIONAL MATCH-REL	1.109	0.303	0.009	1.937	0.088	0.026
SOCIAL RANDOM	0.991	0.387	0.028	-3.033	0.012	-0.045
SOCIAL TOM	0.154	0.889	0.023	-2.132	0.058	-0.020
SOCIAL TOM-RANDOM	-0.363	0.710	-0.015	-4.073	0.003	-0.051
EMOTION FACES	7.379	0.003	0.127	4.654	0.003	0.095
EMOTION SHAPES	2.239	0.058	0.065	1.932	0.079	0.048
EMOTION FACES-SHAPES	15.857	0.003	0.302	12.545	0.003	0.181
WM 2BK_BODY	-1.846	0.100	0.003	-2.885	0.009	-0.036
WM 2BK_FACE	-0.900	0.449	0.002	-2.678	0.012	-0.031
WM 2BK_PLACE	2.718	0.017	0.099	-0.822	0.449	-0.010
WM 2BK_TOOL	-0.823	0.453	0.040	-1.784	0.095	-0.007
WM 0BK_BODY	2.425	0.023	0.059	1.059	0.303	0.031
WM 0BK_FACE	9.014	0.003	0.174	6.231	0.003	0.106
WM 0BK_PLACE	6.796	0.003	0.194	2.375	0.036	0.062
WM 0BK_TOOL	4.895	0.003	0.087	0.231	0.800	0.014
WM 2BK	0.775	0.449	0.061	-1.119	0.345	0.006
WM 0BK	6.993	0.003	0.207	2.210	0.044	0.064
WM 2BK-0BK	-4.629	0.003	-0.049	-4.249	0.003	-0.044

WM BODY	0.708	0.527	0.051	-0.688	0.575	0.009
WM FACE	3.398	0.006	0.155	0.801	0.455	0.060
WM PLACE	7.059	0.003	0.187	1.989	0.048	0.041
WM TOOL	2.154	0.063	0.099	-0.526	0.650	-0.009
WM BODY-AVG	8.045	0.003	0.120	4.803	0.003	0.081
WM FACE-AVG	-1.874	0.081	-0.011	0.385	0.770	0.005
WM PLACE-AVG	1.070	0.364	0.024	0.111	0.916	0.002
WM TOOL-AVG	1.317	0.237	0.011	1.321	0.252	0.044
MOTOR CUE	10.890	0.003	0.218	6.820	0.003	0.112
MOTOR LF	-1.944	0.065	-0.023	-6.651	0.003	-0.090
MOTOR RF	-1.522	0.147	-0.024	-4.113	0.003	-0.076
MOTOR LH	0.737	0.507	0.016	-3.346	0.012	-0.035
MOTOR RH	0.734	0.527	0.032	-2.132	0.046	-0.055
MOTOR T	-11.534	0.003	-0.219	-10.956	0.003	-0.215
MOTOR AVG	1.091	0.357	0.032	-1.954	0.079	-0.028
MOTOR CUE-AVG	15.757	0.003	0.261	13.470	0.003	0.195
MOTOR LF-AVG	-8.918	0.003	-0.223	-17.474	0.003	-0.287
MOTOR LH-AVG	-10.308	0.003	-0.279	-14.821	0.003	-0.248
MOTOR RF-AVG	-9.708	0.003	-0.223	-10.151	0.003	-0.206
MOTOR RH-AVG	-6.365	0.003	-0.155	-9.465	0.003	-0.223
MOTOR T-AVG	-22.767	0.003	-0.377	-14.818	0.003	-0.408
GAMBLING PUNISH	0.142	0.869	0.012	-2.785	0.017	-0.030
GAMBLING REWARD	0.616	0.530	0.006	-2.573	0.023	-0.044
GAMBLING PUNISH-REWARD	2.396	0.025	0.045	-0.016	0.993	0.018

Supplementary Table 5: Paired t-tests with permutation testing (P = 1000) were conducted to compare reconstruction scores between volumetric- and surface-based group-average maps and retest scans in HCP-YA (n = 39). *p*-values were FDR-corrected for four pairwise comparisons across 47 task contrasts (Supplementary Tables 5–6), and effect sizes were reported as Cliff's Delta (δ).

Task Contrast	Linear Model Volume vs. Linear Model Surface			DeepTaskGen vs. BrainSurfCNN		
	t	*p*	*δ*	*t*	*p*	*δ*
LANGUAGE MATH	-20.830	0.003	-0.846	-8.383	0.003	-0.345
LANGUAGE STORY	-13.858	0.003	-0.528	-3.686	0.003	-0.168
LANGUAGE MATH-STORY	-35.633	0.003	-0.925	-21.077	0.003	-0.532
RELATIONAL MATCH	-68.981	0.003	-0.967	-15.381	0.003	-0.385
RELATIONAL REL	-57.475	0.003	-0.955	-19.215	0.003	-0.432
RELATIONAL MATCH-REL	-8.306	0.003	-0.300	-2.465	0.023	-0.059
SOCIAL RANDOM	-39.734	0.003	-0.928	-11.258	0.003	-0.365
SOCIAL TOM	-51.505	0.003	-0.988	-14.498	0.003	-0.467
SOCIAL TOM-RANDOM	-24.190	0.003	-0.813	-5.335	0.003	-0.161
EMOTION FACES	-30.019	0.003	-0.799	-6.825	0.003	-0.160
EMOTION SHAPES	-29.832	0.003	-0.741	-8.487	0.003	-0.197
EMOTION FACES-SHAPES	-24.514	0.003	-0.819	0.657	0.575	0.019
WM 2BK_BODY	-30.465	0.003	-0.822	-15.777	0.003	-0.406
WM 2BK_FACE	-38.978	0.003	-0.884	-15.312	0.003	-0.437
WM 2BK_PLACE	-43.043	0.003	-0.901	-13.607	0.003	-0.357
WM 2BK_TOOL	-30.233	0.003	-0.761	-16.192	0.003	-0.396
WM 0BK_BODY	-27.220	0.003	-0.719	-7.732	0.003	-0.183
WM 0BK_FACE	-30.147	0.003	-0.782	-5.813	0.003	-0.139
WM 0BK_PLACE	-52.736	0.003	-0.916	-8.937	0.003	-0.266
WM 0BK_TOOL	-34.323	0.003	-0.867	-12.172	0.003	-0.270
WM 2BK	-43.954	0.003	-0.867	-18.020	0.003	-0.575
WM 0BK	-47.574	0.003	-0.904	-11.490	0.003	-0.414
WM 2BK-0BK	-15.014	0.003	-0.475	-11.240	0.003	-0.207
WM BODY	-33.976	0.003	-0.819	-14.737	0.003	-0.382

WM FACE	-44.003	0.003	-0.892	-12.456	0.003	-0.435
WM PLACE	-56.097	0.003	-0.964	-13.000	0.003	-0.412
WM TOOL	-39.808	0.003	-0.863	-17.850	0.003	-0.399
WM BODY-AVG	-18.699	0.003	-0.667	1.521	0.173	0.053
WM FACE-AVG	-26.361	0.003	-0.633	-9.219	0.003	-0.127
WM PLACE-AVG	-27.803	0.003	-0.698	-3.314	0.003	-0.057
WM TOOL-AVG	-15.833	0.003	-0.379	0.396	0.690	0.003
MOTOR CUE	-26.948	0.003	-0.763	-2.650	0.009	-0.062
MOTOR LF	-22.360	0.003	-0.658	-5.193	0.006	-0.144
MOTOR RF	-20.721	0.003	-0.699	-3.569	0.009	-0.119
MOTOR LH	-20.105	0.003	-0.553	-3.624	0.003	-0.097
MOTOR RH	-20.009	0.003	-0.600	-2.583	0.028	-0.059
MOTOR T	-25.687	0.003	-0.683	-10.448	0.003	-0.211
MOTOR AVG	-25.023	0.003	-0.673	-3.583	0.006	-0.080
MOTOR CUE-AVG	-25.178	0.003	-0.744	2.220	0.053	0.074
MOTOR LF-AVG	-23.129	0.003	-0.783	-10.429	0.003	-0.265
MOTOR LH-AVG	-18.211	0.003	-0.616	-7.863	0.003	-0.265
MOTOR RF-AVG	-26.398	0.003	-0.753	-10.437	0.003	-0.248
MOTOR RH-AVG	-15.229	0.003	-0.496	-5.711	0.003	-0.178
MOTOR T-AVG	-30.060	0.003	-0.670	-15.051	0.003	-0.341
GAMBLING PUNISH	-41.406	0.003	-0.895	-11.502	0.003	-0.375
GAMBLING REWARD	-54.716	0.003	-0.937	-14.634	0.003	-0.417
GAMBLING PUNISH-REWARD	-6.491	0.003	-0.277	-2.596	0.025	-0.043

Supplementary Table 6: Paired t-tests with permutation testing (P = 1000) were conducted to compare reconstruction scores between volumetric- and surface-based linear and deep-learning based models (DeepTaskGen vs. BrainVolCNN[4]) in HCP-YA (n = 39). *p*-values were FDR-corrected for four pairwise comparisons across 47 task contrasts (Supplementary Tables 5–6), and effect sizes were reported as Cliff's Delta (δ).

Task Contrast	Retest Volume vs. Retest Surface			Linear Model Volume vs. Linear Model Surface			DeepTaskGen vs. BrainSurfCNN		
	t	*p*	*δ*	*t*	*p*	*δ*	*t*	*p*	*δ*
LANGUAGE MATH	-2.910	0.007	-0.064	-14.685	0.002	-0.992	-17.621	0.002	-0.988
LANGUAGE STORY	-1.987	0.071	-0.052	-10.684	0.002	-0.901	-10.127	0.002	-0.836
LANGUAGE MATH-STORY	1.921	0.055	0.040	-17.819	0.002	-1.000	-24.993	0.002	-1.000
RELATIONAL MATCH	-2.734	0.018	-0.032	-24.778	0.002	-0.997	-33.634	0.002	-1.000
RELATIONAL REL	-0.827	0.456	-0.007	-22.090	0.002	-0.997	-33.435	0.002	-1.000
RELATIONAL MATCH-REL	1.204	0.244	0.030	-5.999	0.002	-0.648	-6.547	0.002	-0.609
SOCIAL RANDOM	-3.331	0.005	-0.055	-16.152	0.002	-0.989	-23.397	0.002	-0.999
SOCIAL TOM	-2.687	0.018	-0.059	-20.335	0.002	-0.984	-31.001	0.002	-1.000
SOCIAL TOM-RANDOM	-3.635	0.002	-0.064	-11.992	0.002	-0.967	-11.964	0.002	-0.913
EMOTION FACES	-1.242	0.244	-0.018	-17.771	0.002	-0.986	-21.113	0.002	-0.992
EMOTION SHAPES	-0.271	0.809	-0.001	-15.355	0.002	-0.947	-17.792	0.002	-0.940
EMOTION FACES-SHAPES	2.144	0.038	0.041	-16.280	0.002	-0.980	-19.731	0.002	-0.996
WM 2BK_BODY	-1.882	0.079	-0.024	-12.852	0.002	-0.945	-18.707	0.002	-0.932
WM 2BK_FACE	-2.932	0.007	-0.041	-16.464	0.002	-0.974	-24.670	0.002	-0.988
WM 2BK_PLACE	-3.582	0.002	-0.052	-18.493	0.002	-0.993	-23.609	0.002	-1.000
WM 2BK_TOOL	-1.733	0.087	-0.018	-14.986	0.002	-0.945	-17.903	0.002	-0.938
WM 0BK_BODY	-1.931	0.071	-0.027	-15.586	0.002	-0.941	-15.623	0.002	-0.947
WM 0BK_FACE	-0.387	0.715	-0.023	-13.547	0.002	-0.937	-18.289	0.002	-0.938
WM 0BK_PLACE	-3.591	0.005	-0.077	-21.145	0.002	-0.992	-24.380	0.002	-1.000
WM 0BK_TOOL	-3.910	0.005	-0.066	-17.939	0.002	-0.976	-20.801	0.002	-0.978
WM 2BK	-3.071	0.002	-0.040	-17.725	0.002	-0.984	-26.007	0.002	-0.979
WM 0BK	-5.393	0.002	-0.093	-20.833	0.002	-0.984	-27.549	0.002	-1.000
WM 2BK-0BK	-1.953	0.079	-0.027	-9.008	0.002	-0.740	-9.817	0.002	-0.712

WM BODY	-2.225	0.045	-0.039	-15.666	0.002	-0.950	-20.257	0.002	-0.954
WM FACE	-3.665	0.002	-0.049	-17.091	0.002	-0.974	-26.335	0.002	-1.000
WM PLACE	-4.680	0.002	-0.069	-21.529	0.002	-1.000	-29.633	0.002	-1.000
WM TOOL	-3.435	0.005	-0.048	-18.042	0.002	-0.968	-23.238	0.002	-0.971
WM BODY-AVG	-0.359	0.761	0.015	-9.362	0.002	-0.895	-11.697	0.002	-0.815
WM FACE-AVG	1.998	0.055	0.024	-11.627	0.002	-0.933	-12.938	0.002	-0.955
WM PLACE-AVG	-0.601	0.531	-0.002	-12.729	0.002	-0.882	-10.945	0.002	-0.836
WM TOOL-AVG	2.080	0.051	0.043	-9.284	0.002	-0.822	-8.103	0.002	-0.740
MOTOR CUE	-2.015	0.071	-0.043	-19.121	0.002	-0.988	-22.296	0.002	-0.975
MOTOR LF	-4.085	0.002	-0.065	-10.167	0.002	-0.801	-11.024	0.002	-0.862
MOTOR RF	-1.310	0.196	-0.049	-11.075	0.002	-0.874	-9.662	0.002	-0.773
MOTOR LH	-3.093	0.005	-0.043	-9.171	0.002	-0.813	-9.923	0.002	-0.817
MOTOR RH	-2.046	0.057	-0.043	-10.322	0.002	-0.804	-9.151	0.002	-0.834
MOTOR T	-3.595	0.009	-0.085	-11.799	0.002	-0.859	-11.939	0.002	-0.899
MOTOR AVG	-2.423	0.034	-0.044	-12.463	0.002	-0.880	-12.206	0.002	-0.922
MOTOR CUE-AVG	-1.421	0.174	-0.016	-17.390	0.002	-0.970	-19.228	0.002	-0.955
MOTOR LF-AVG	-5.248	0.002	-0.136	-7.388	0.002	-0.719	-6.107	0.002	-0.528
MOTOR LH-AVG	-4.564	0.002	-0.089	-9.881	0.002	-0.809	-6.902	0.002	-0.652
MOTOR RF-AVG	-3.847	0.002	-0.089	-6.290	0.002	-0.677	-5.351	0.002	-0.519
MOTOR RH-AVG	-3.510	0.002	-0.094	-8.459	0.002	-0.620	-5.892	0.002	-0.565
MOTOR T-AVG	-5.615	0.002	-0.166	-8.189	0.002	-0.702	-7.477	0.002	-0.737
GAMBLING PUNISH	-1.723	0.106	-0.015	-17.903	0.002	-0.995	-23.042	0.002	-1.000
GAMBLING REWARD	-2.233	0.018	-0.037	-23.244	0.002	-1.000	-27.571	0.002	-1.000
GAMBLING PUNISH-REWARD	-0.533	0.599	0.026	-4.637	0.002	-0.583	-4.596	0.002	-0.439

Supplementary Table 7: Paired t-tests with permutation testing (P = 1000) were conducted to compare diagonality index scores between volumetric- and surface-based retest scans, linear model[6], and deep learning models BrainVolCNN[4] in HCP-YA (n = 39). The diagonality index of group-average maps was 0 for both brain representations. *p*-values were FDR-corrected for three pairwise comparisons across 47 task contrasts, and effect sizes were expressed as Cliff's Delta (δ).

Task Contrast	Group Average Volume vs. Group Average Surface			Retest Volume vs. Retest Surface		
	t	*p*	*δ*	*t*	*p*	*δ*
LANGUAGE MATH	6.072	0.002	0.235	6.571	0.002	0.115
LANGUAGE STORY	5.140	0.002	0.119	4.683	0.002	0.073
LANGUAGE MATH-STORY	-0.686	0.497	-0.034	1.975	0.045	0.039
RELATIONAL MATCH	7.927	0.002	0.083	8.044	0.002	0.091
RELATIONAL REL	6.315	0.002	0.094	7.295	0.002	0.087
RELATIONAL MATCH-REL	4.172	0.002	0.108	7.506	0.002	0.137
SOCIAL RANDOM	3.169	0.002	0.051	-1.783	0.121	-0.028
SOCIAL TOM	7.464	0.002	0.145	4.403	0.002	0.081
SOCIAL TOM-RANDOM	3.183	0.002	0.053	6.288	0.002	0.070
EMOTION FACES	9.369	0.002	0.152	18.102	0.002	0.207
EMOTION SHAPES	6.289	0.002	0.090	12.499	0.002	0.124
EMOTION FACES-SHAPES	15.713	0.002	0.321	21.783	0.002	0.289
WM 2BK_BODY	11.555	0.002	0.161	11.470	0.002	0.157
WM 2BK_FACE	12.437	0.002	0.235	11.189	0.002	0.140
WM 2BK_PLACE	10.543	0.002	0.172	10.590	0.002	0.114
WM 2BK_TOOL	12.004	0.002	0.166	12.245	0.002	0.116
WM 0BK_BODY	9.134	0.002	0.130	12.968	0.002	0.127
WM 0BK_FACE	14.455	0.002	0.240	13.531	0.002	0.215
WM 0BK_PLACE	11.559	0.002	0.250	11.527	0.002	0.151
WM 0BK_TOOL	9.597	0.002	0.151	11.205	0.002	0.118
WM 2BK	15.933	0.002	0.281	12.294	0.002	0.157
WM 0BK	19.480	0.002	0.249	12.316	0.002	0.140
WM 2BK-0BK	1.881	0.087	0.023	2.917	0.009	0.026

WM BODY	12.973	0.002	0.177	10.824	0.002	0.149
WM FACE	17.131	0.002	0.321	12.380	0.002	0.153
WM PLACE	13.622	0.002	0.279	12.193	0.002	0.127
WM TOOL	12.895	0.002	0.194	13.356	0.002	0.127
WM BODY-AVG	5.107	0.002	0.116	10.752	0.002	0.141
WM FACE-AVG	-2.773	0.011	-0.041	3.715	0.002	0.040
WM PLACE-AVG	2.796	0.014	0.041	7.032	0.002	0.087
WM TOOL-AVG	1.377	0.201	0.026	6.838	0.002	0.114
MOTOR CUE	13.241	0.002	0.358	9.567	0.002	0.140
MOTOR LF	-5.851	0.002	-0.131	-4.535	0.002	-0.057
MOTOR RF	0.378	0.693	0.012	-1.371	0.217	-0.012
MOTOR LH	-2.848	0.002	-0.061	-3.348	0.002	-0.030
MOTOR RH	0.506	0.660	-0.003	-1.415	0.187	-0.015
MOTOR T	-13.037	0.002	-0.299	-7.860	0.002	-0.147
MOTOR AVG	-1.544	0.155	-0.061	-1.310	0.219	-0.007
MOTOR CUE-AVG	17.306	0.002	0.323	16.557	0.002	0.228
MOTOR LF-AVG	-11.275	0.002	-0.361	-10.041	0.002	-0.336
MOTOR LH-AVG	-9.416	0.002	-0.319	-12.236	0.002	-0.304
MOTOR RF-AVG	-11.988	0.002	-0.332	-9.933	0.002	-0.281
MOTOR RH-AVG	-9.205	0.002	-0.227	-10.294	0.002	-0.261
MOTOR T-AVG	-14.693	0.002	-0.517	-9.044	0.002	-0.395
GAMBLING PUNISH	16.035	0.002	0.264	12.714	0.002	0.144
GAMBLING REWARD	12.575	0.002	0.179	12.042	0.002	0.164
GAMBLING PUNISH-REWARD	-3.084	0.005	-0.099	4.519	0.002	0.173

Supplementary Table 8: Paired t-tests with permutation testing (P = 1000) were conducted to compare Dice-AUC scores between volumetric- and surface-based group-average maps and retest scans in HCP-YA (n = 39). *p*-values were FDR-corrected for four pairwise comparisons across 47 task contrasts (Supplementary Tables 8–9), and effect sizes were reported as Cliff's Delta (δ).

Task Contrast	Linear Regression Volume vs. Linear Regression Surface			DeepTaskGen vs. BrainSurfCNN		
	t	*p*	*δ*	*t*	*p*	*δ*
LANGUAGE MATH	-9.154	0.002	-0.377	-0.826	0.468	-0.028
LANGUAGE STORY	-8.519	0.002	-0.340	0.141	0.871	0.006
LANGUAGE MATH-STORY	-22.065	0.002	-0.817	-14.972	0.002	-0.423
RELATIONAL MATCH	-28.385	0.002	-0.766	-10.189	0.002	-0.250
RELATIONAL REL	-24.271	0.002	-0.721	-10.924	0.002	-0.224
RELATIONAL MATCH-REL	-5.139	0.002	-0.218	-0.706	0.486	0.012
SOCIAL RANDOM	-28.763	0.002	-0.844	-8.268	0.002	-0.202
SOCIAL TOM	-24.257	0.002	-0.855	-7.902	0.002	-0.262
SOCIAL TOM-RANDOM	-12.872	0.002	-0.487	-1.098	0.302	-0.023
EMOTION FACES	-20.610	0.002	-0.508	-0.857	0.431	-0.003
EMOTION SHAPES	-17.058	0.002	-0.517	-3.201	0.002	-0.048
EMOTION FACES-SHAPES	-12.131	0.002	-0.486	2.112	0.051	0.101
WM 2BK_BODY	-13.897	0.002	-0.492	-4.791	0.002	-0.097
WM 2BK_FACE	-19.513	0.002	-0.604	-5.292	0.002	-0.158
WM 2BK_PLACE	-16.472	0.002	-0.686	-4.914	0.002	-0.181
WM 2BK_TOOL	-15.150	0.002	-0.445	-7.277	0.002	-0.145
WM 0BK_BODY	-13.334	0.002	-0.357	-3.166	0.007	-0.053
WM 0BK_FACE	-11.471	0.002	-0.457	-1.300	0.218	-0.039
WM 0BK_PLACE	-20.601	0.002	-0.742	-3.613	0.005	-0.116
WM 0BK_TOOL	-16.841	0.002	-0.502	-4.456	0.002	-0.097
WM 2BK	-23.197	0.002	-0.738	-7.911	0.002	-0.282
WM 0BK	-22.487	0.002	-0.723	-5.545	0.002	-0.172
WM 2BK-0BK	-9.064	0.002	-0.325	-6.034	0.002	-0.119

WM BODY	-18.383	0.002	-0.584	-6.424	0.002	-0.140
WM FACE	-19.369	0.002	-0.712	-4.719	0.002	-0.198
WM PLACE	-18.264	0.002	-0.784	-4.331	0.002	-0.191
WM TOOL	-17.883	0.002	-0.600	-6.625	0.002	-0.187
WM BODY-AVG	-9.898	0.002	-0.361	1.775	0.097	0.048
WM FACE-AVG	-14.409	0.002	-0.432	-7.108	0.002	-0.090
WM PLACE-AVG	-10.870	0.002	-0.423	-0.899	0.392	-0.043
WM TOOL-AVG	-7.088	0.002	-0.290	0.791	0.431	0.010
MOTOR CUE	-19.226	0.002	-0.661	1.011	0.311	0.056
MOTOR LF	-15.352	0.002	-0.627	-8.064	0.002	-0.201
MOTOR RF	-13.751	0.002	-0.537	-3.675	0.005	-0.156
MOTOR LH	-13.211	0.002	-0.504	-6.331	0.002	-0.148
MOTOR RH	-11.582	0.002	-0.490	-3.005	0.005	-0.120
MOTOR T	-15.127	0.002	-0.550	-7.521	0.002	-0.245
MOTOR AVG	-14.136	0.002	-0.662	-5.226	0.002	-0.201
MOTOR CUE-AVG	-15.286	0.002	-0.566	7.750	0.002	0.211
MOTOR LF-AVG	-14.164	0.002	-0.586	-12.412	0.002	-0.396
MOTOR LH-AVG	-13.959	0.002	-0.469	-10.240	0.002	-0.336
MOTOR RF-AVG	-12.657	0.002	-0.382	-12.442	0.002	-0.383
MOTOR RH-AVG	-9.295	0.002	-0.298	-10.539	0.002	-0.277
MOTOR T-AVG	-12.289	0.002	-0.652	-3.788	0.002	-0.262
GAMBLING PUNISH	-22.098	0.002	-0.749	-2.557	0.025	-0.091
GAMBLING REWARD	-19.653	0.002	-0.631	-3.633	0.005	-0.106
GAMBLING PUNISH-REWARD	-4.061	0.002	-0.130	-3.243	0.002	-0.099

Supplementary Table 9: Paired t-tests with permutation testing (P = 1000) were conducted to compare Dice-AUC scores between volumetric- and surface-based linear[6] and deep-learning based models (DeepTaskGen vs. BrainSurfCNN[4]) in HCP-YA (n = 39). *p*-values were FDR-corrected for four pairwise comparisons across 47 task contrasts (Supplementary Tables 8–9), and effect sizes were reported as Cliff's Delta (δ).

Task Contrast	Group Average	Retest Scans	Linear Model	BrainSurfCNN
LANGUAGE MATH	0	0.386	0.435	0.462
LANGUAGE STORY	0	0.314	0.403	0.418
LANGUAGE MATH-STORY	0	0.694	0.668	0.702
RELATIONAL MATCH	0	0.717	0.693	0.743
RELATIONAL REL	0	0.736	0.706	0.758
RELATIONAL MATCH-REL	0	0.111	0.201	0.174
SOCIAL RANDOM	0	0.716	0.661	0.712
SOCIAL TOM	0	0.754	0.697	0.745
SOCIAL TOM-RANDOM	0	0.266	0.415	0.392
EMOTION FACES	0	0.480	0.542	0.586
EMOTION SHAPES	0	0.397	0.539	0.544
EMOTION FACES-SHAPES	0	0.422	0.420	0.482
WM 2BK_BODY	0	0.524	0.571	0.631
WM 2BK_FACE	0	0.555	0.571	0.653
WM 2BK_PLACE	0	0.579	0.630	0.689
WM 2BK_TOOL	0	0.516	0.557	0.600
WM 0BK_BODY	0	0.367	0.492	0.543
WM 0BK_FACE	0	0.368	0.492	0.558
WM 0BK_PLACE	0	0.539	0.640	0.693
WM 0BK_TOOL	0	0.449	0.597	0.648
WM 2BK	0	0.688	0.659	0.732
WM 0BK	0	0.636	0.659	0.724
WM 2BK-0BK	0	0.277	0.408	0.431
WM BODY	0	0.566	0.576	0.637
WM FACE	0	0.605	0.588	0.673
WM PLACE	0	0.632	0.681	0.743
WM TOOL	0	0.598	0.632	0.693

WM BODY-AVG	0	0.160	0.273	0.280
WM FACE-AVG	0	0.254	0.349	0.371
WM PLACE-AVG	0	0.231	0.368	0.396
WM TOOL-AVG	0	0.075	0.206	0.134
MOTOR CUE	0	0.469	0.570	0.591
MOTOR LF	0	0.305	0.355	0.418
MOTOR RF	0	0.259	0.363	0.359
MOTOR LH	0	0.275	0.361	0.388
MOTOR RH	0	0.277	0.342	0.383
MOTOR T	0	0.303	0.415	0.446
MOTOR AVG	0	0.375	0.436	0.500
MOTOR CUE-AVG	0	0.554	0.569	0.596
MOTOR LF-AVG	0	0.122	0.158	0.107
MOTOR LH-AVG	0	0.156	0.273	0.239
MOTOR RF-AVG	0	0.086	0.158	0.103
MOTOR RH-AVG	0	0.139	0.196	0.158
MOTOR T-AVG	0	0.267	0.275	0.233
GAMBLING PUNISH	0	0.604	0.630	0.685
GAMBLING REWARD	0	0.629	0.656	0.710
GAMBLING PUNISH-REWARD	0	0.018	0.055	0.045

Supplementary Table 10: The fingerprinting scores of surface-based baselines and generative methods (e.g., linear model[6] and BrainSurfCNN[4]) performed on the HCP-YA dataset. The fingerprinting scores of the volumetric-based methods are given in Supplementary Table 2.

Task Contrast	DeepTaskGen Fine-tuned vs. Linear Model			DeepTaskGen Fine-tuned vs. DeepTaskGen Non-Fine-tuned		
	t	*p*	*δ*	*t*	*p*	*δ*
EMOTION FACES-SHAPES	.018	.995	-	**-11.059**	**.004**	**-.624**
GAMBLING REWARD	**10.225**	**.004**	**.340**	**3.675**	**.005**	**.142**

Supplementary Table 11: Paired t-tests with permutation testing ($P = 1000$) were performed to compare the reconstruction performance of the fine-tuned DeepTaskGen model with the linear model and the non-fine-tuned DeepTaskGen model on HCP-D. FDR corrected (across model and contrasts) significant tests are highlighted. The sample size for this comparison was 64. Cliff's Delta (δ) was used to measure effect size.

Task Contrast	DeepTaskGen Fine-tuned vs. Linear Model			DeepTaskGen Fine-tuned vs. DeepTaskGen Non-Fine-tuned		
	t	*p*	*δ*	*t*	*p*	*δ*
EMOTION FACES-SHAPES	**3.998**	**.003**	**.347**	**4.392**	**.003**	**.333**
GAMBLING REWARD	1.362	.200	-	**5.743**	**.003**	**.356**

Supplementary Table 12: Paired t-tests with permutation testing ($P = 1000$) were performed to compare the Diagonality Index scores of the fine-tuned DeepTaskGen model with the linear model and the non-fine-tuned DeepTaskGen model on HCP-D. FDR corrected (across model and contrasts) significant tests are highlighted. The sample size for this comparison was 64. Cliff's Delta (δ) was used to measure effect size.

Task Contrast	DeepTaskGen Fine-tuned vs. Linear Model			DeepTaskGen Fine-tuned vs. DeepTaskGen Non-Fine-tuned		
	t	*p*	*δ*	*t*	*p*	*δ*
EMOTION FACES-SHAPES	**-2.738**	**.004**	**-.211**	**-8.955**	**.003**	**-.559**
GAMBLING REWARD	**7.231**	**.003**	**.208**	**5.876**	**.003**	**.145**

Supplementary Table 13: Paired t-tests with permutation testing ($P = 1000$) were performed to compare the Dice AUC of the fine-tuned DeepTaskGen model with the linear model and the non-fine-tuned DeepTaskGen model on HCP-D. FDR corrected (across model and contrasts) significant tests are highlighted. The sample size for this comparison was 64. Cliff's Delta (δ) was used to measure effect size.

Task Contrast	DeepTaskGen Fine-tuned	DeepTaskGen Non-Fine-tuned	Linear Model
EMOTION FACES-SHAPES	.137	**.284**	.274
GAMBLING REWARD	.141	.069	**.222**

Supplementary Table 14: The fingerprinting score for various models. The table presents the results obtained from analyses performed on the HCP-D dataset. The highest fingerprinting score for each task contrast is highlighted, indicating the method that achieved the best performance in differentiating between individuals based on their task contrast maps.

Measure	UK Biobank variable IDs
Age	21003
Sex	31
Fluid Intelligence	20016
Dominant Hand Strength	46, 47, 1707
Overall Health	2178
Alcohol Use Frequency	1558
Weekly Beer Intake	1588
Depression	20002-0.1286
Hypertension	20002-0.1065
Neuroticism	20127
PHQ-9	20507, 20508, 20510, 20511, 20513, 20514, 20517, 20518, 20519
GAD-7	20505, 20506, 20509, 20512, 20515, 20516, 20520
RDS-4	2050, 2060, 2070, 2080

Supplementary Table 15: Subjects’ measures available in the UK Biobank. Abbreviations: PHQ-9: Patient Health Questionnaire-9; GAD-7: General Anxiety Disorder-7; RDS-4: Recent Depressive Symptoms-4.

Task Contrast		Age		Sex		Fluid Intelligence		Grip Strength		Overall Health		Alcohol Use Frequency		Beer Use Frequency	
		μ_{cv}	p	μ_{cv}	p	μ_{cv}	p	μ_{cv}	p	μ_{cv}	p	μ_{cv}	p	μ_{cv}	p
Actual	RESTING STATE	**.413**	**.001**	**.781**	**.001**	**.113**	**.002**	**.357**	**.001**	.006	.418	.041	.072	**.131**	**.001**
	EMOTION FACES-SHAPES	**.378**	**.001**	**.704**	**.001**	**.118**	**.002**	**.364**	**.001**	**.066**	**.013**	-.016	.727	.043	.107
Predicted	EMOTION FACES-SHAPES	**.459**	**.001**	**.837**	**.001**	**.094**	**.003**	**.349**	**.001**	.037	.114	**.072**	**.008**	**.212**	**.001**
	GAMBLING REWARD	**.452**	**.001**	**.828**	**.001**	**.054**	**.040**	**.401**	**.001**	.015	.329	.044	.076	**.168**	**.001**
	LANGUAGE MATH-STORY	**.446**	**.001**	**.817**	**.001**	**.094**	**.001**	**.418**	**.001**	.017	.302	**.112**	**.001**	**.181**	**.001**
	MOTOR AVG	**.538**	**.001**	**.836**	**.001**	**.076**	**.009**	**.356**	**.001**	.013	.308	**.073**	**.010**	**.182**	**.001**
	RELATIONAL REL	**.475**	**.001**	**.820**	**.001**	**.056**	**.037**	**.450**	**.001**	.028	.188	**.055**	**.033**	**.160**	**.001**
	SOCIAL TOM-RANDOM	**.476**	**.001**	**.827**	**.001**	**.111**	**.002**	**.349**	**.001**	.035	.135	**.078**	**.007**	**.190**	**.001**
	WM 2BK-0BK	**.487**	**.001**	**.827**	**.001**	**.067**	**.012**	**.429**	**.001**	-.014	.676	**.053**	**.025**	**.182**	**.001**

Supplementary Table 16. Prediction performances for demographic, cognitive and behavioral measures. Out-of-sample performance was evaluated using a 5-fold cross-validation framework and permutation testing. $\boldsymbol{\mu_{cv}}$ represents the mean CV scores. Balanced accuracy was used for sex, while Pearson's correlation coefficient assessed the remaining variables. Significant predictions ($p < .05$) are highlighted. Comparisons between actual and synthetic data, which achieved significant predictions, were made using permutation testing ($P = 1000$), and the results are presented in Supplementary Tables 18-22.

Task Contrast		Depression		Hypertension		GAD-7		RDS-4		PHQ-9		Neuroticism	
		μ_{cv}	p	μ_{cv}	p	μ_{cv}	p	μ_{cv}	p	μ_{cv}	p	μ_{cv}	p
Actual	RESTING STATE	.504	.368	.508	.250	**.083**	**.008**	.018	.289	.050	.083	.032	.152
	EMOTION FACES-SHAPES	.502	.345	**.526**	**.017**	.031	.183	.012	.328	.008	.399	.048	.083
Predicted	EMOTION FACES-SHAPES	.513	.405	.517	.087	.013	.377	.029	.166	**.062**	**.038**	.015	.314
	GAMBLING REWARD	.502	.405	**.553**	**.001**	.043	.118	.033	.150	.016	.342	.035	.132
	LANGUAGE MATH-STORY	.486	.945	**.539**	**.003**	-.002	.515	.018	.262	.016	.311	.019	.282
	MOTOR AVG	.502	.409	**.524**	**.030**	**.091**	**.005**	.016	.188	**.085**	**.008**	.026	.224
	RELATIONAL REL	.509	.155	**.535**	**.005**	.053	.065	**.052**	**.049**	.031	.191	.047	.075
	SOCIAL TOM-RANDOM	.510	.104	.512	.148	.043	.105	-.004	.536	.039	.163	.019	.296
	WM 2BK-0BK	.508	.174	**.539**	**.001**	.034	.159	.041	.009	**.074**	**.015**	-.018	.711

Supplementary Table 17. Prediction performances for physical and mental health measures. Out-of-sample performance was evaluated using a 5-fold cross-validation framework and permutation testing. $\boldsymbol{\mu_{cv}}$ represents the mean CV scores. Balanced accuracy was used for depression and hypertension classification, while Pearson's correlation coefficient assessed the remaining variables. Significant predictions ($p < .05$) are highlighted. Comparisons between actual and synthetic data, which achieved significant predictions, were made using permutation testing ($P = 1000$), and the results are presented in Supplementary Tables 23,24.

Task Contrast		vs. Actual EMOTION FACES-SHAPES			vs. Resting State Connectome		
		t	*p*	*δ*	*t*	*p*	*δ*
Predicted	EMOTION FACES-SHAPES	3.94	.050	-	2.55	.076	-
	GAMBLING REWARD	3.79	.050	-	2.38	.097	-
	LANGUAGE MATH-STORY	**6.51**	**.030**	**1.0**	2.92	.066	-
	MOTOR AVG	**6.59**	**.030**	**1.0**	**5.52**	**.038**	**1.0**
	RELATIONAL REL	**5.14**	**.038**	**1.0**	3.97	.504	-
	SOCIAL TOM-RANDOM	3.43	.050	-	2.45	.076	-
	WM 2BK-0BK	**5.29**	**.038**	**1.0**	4.14	.050	-
Actual	Resting State Connectome	**10.19**	**.030**	**.68**			

Supplementary Table 18: Age prediction. The results of paired t-tests with permutation testing ($P = 1000$), comparing the brain age prediction performance of predicted task contrast maps, actual EMOTION FACES-SHAPES task contrast maps, and resting-state connectome data from the UK Biobank dataset. Significant differences ($p < .05, FDR\ corrected$) are highlighted in bold. Cliff's Delta (δ) was used to measure effect size.

Task Contrast		vs. Actual EMOTION FACES-SHAPES			vs. Resting State Connectome		
		t	*p*	*δ*	*t*	*p*	*δ*
Predicted	EMOTION FACES-SHAPES	**9.08**	**.012**	**1.0**	**6.79**	**.009**	**1.0**
	GAMBLING REWARD	**6.08**	**.012**	**1.0**	**6.63**	**.012**	**.84**
	LANGUAGE MATH-STORY	**8.21**	**.007**	**1.0**	**3.67**	**.034**	**.84**
	MOTOR AVG	**9.77**	**.007**	**1.0**	**3.53**	**.030**	**.92**
	RELATIONAL REL	**6.55**	**.012**	**1.0**	**12.8**	**.007**	**.84**
	SOCIAL TOM-RANDOM	**7.14**	**.012**	**1.0**	**4.02**	**.029**	**.84**
	WM 2BK-0BK	**9.26**	**.007**	**1.0**	2.65	.055	-
Actual	Resting State Connectome	**3.76**	**.030**	**1.0**			

Supplementary Table 19: Sex classification. The results of paired t-tests with permutation testing ($P = 1000$), comparing the sex classification performance of predicted task contrast maps, actual EMOTION FACES-SHAPES task contrast maps, and resting-state connectome data from the UK Biobank dataset. Significant differences ($p < .05, FDR\ corrected$) are highlighted in bold. Cliff's Delta (δ) was used to measure effect size.

Task Contrast		vs. Actual EMOTION FACES-SHAPES		vs. Resting State Connectome	
		t	*p*	*t*	*p*
Predicted	EMOTION FACES-SHAPES	-.78	.433	-.91	.433
	GAMBLING REWARD	-2.23	.378	-2.71	.378
	LANGUAGE MATH-STORY	-.63	.433	-.64	.433
	MOTOR AVG	-1.19	.433	-1.31	.433
	RELATIONAL REL	-2.54	.378	-2.22	.378
	SOCIAL TOM-RANDOM	-.47	.458	-.06	.495
	WM 2BK-0BK	-1.92	.402	-1.66	.407
Actual	Resting State Connectome	-.11	.495		

Supplementary Table 20: Fluid intelligence prediction. The results of paired t-tests with permutation testing $(P = 1000)$, comparing the fluid intelligence prediction performance of predicted task contrast maps, actual EMOTION FACES-SHAPES task contrast maps, and resting-state connectome data from the UK Biobank dataset. No significant difference was found $(p > .05, FDR\ corrected)$.

Task Contrast		vs. Actual EMOTION FACES-SHAPES		vs. Resting State Connectome	
		t	*p*	*t*	*p*
Predicted	EMOTION FACES-SHAPES	-.31	.495	-.58	.495
	GAMBLING REWARD	1.09	.466	2.20	.466
	LANGUAGE MATH-STORY	1.64	.466	1.65	.466
	MOTOR AVG	-.12	.495	-.02	.495
	RELATIONAL REL	1.64	.466	3.42	.466
	SOCIAL TOM-RANDOM	-.32	.495	-.22	.495
	WM 2BK-0BK	1.13	.466	2.40	.466
Actual	Resting State Connectome	-.13	.495		

Supplementary Table 21: Dominant hand grip strength prediction. The results of paired t-tests with permutation testing $(P = 1000)$, comparing the dominant hand grip strength prediction performance of predicted task contrast maps, actual EMOTION FACES-SHAPES task contrast maps, and resting-state connectome data from the UK Biobank dataset. No significant difference was found $(p > .05, FDR\ corrected)$.

Task Contrast		vs. Actual EMOTION FACES-SHAPES		vs. Resting State Connectome	
		t	*p*	*t*	*p*
Predicted	EMOTION FACES-SHAPES	-	-	4.38	.050
	GAMBLING REWARD	-	-	1.33	.241
	LANGUAGE MATH-STORY	-	-	1.36	.250
	MOTOR AVG	-	-	1.18	.252
	RELATIONAL REL	-	-	.94	.252
	SOCIAL TOM-RANDOM	-	-	1.50	.241
	WM 2BK-0BK	-	-	1.91	.226
Actual	Resting State Connectome	-	-	-	-

Supplementary Table 22: Weekly beer intake prediction. The results of paired t-tests with permutation testing $(P = 1000)$, comparing the weekly beer intake prediction performance of predicted task contrast maps, actual EMOTION FACES-SHAPES task contrast maps, and resting-state connectome data from the UK Biobank dataset. Actual EMOTION FACES-SHAPES did not survive the permutation test. No significant difference was found $(p > .05, FDR\ corrected)$.

Task Contrast		vs. Actual EMOTION FACES-SHAPES		vs. Resting State Connectome	
		t	*p*	*t*	*p*
Predicted	EMOTION FACES-SHAPES	-.51	.418	-	-
	GAMBLING REWARD	1.95	.230	-	-
	LANGUAGE MATH-STORY	1.41	.268	-	-
	MOTOR AVG	-.21	.452	-	-
	RELATIONAL REL	.42	.418	-	-
	SOCIAL TOM-RANDOM	-3.39	.206	-	-
	WM 2BK-0BK	.96	.348	-	-
Actual	Resting State Connectome	-	-	-	-

Supplementary Table 23: Hypertension diagnosis classification. The results of paired t-tests with permutation testing $(P = 1000)$, comparing the hypertension diagnosis classification performance of predicted task contrast maps, actual EMOTION FACES-SHAPES task contrast maps, and resting-state connectome data from the UK Biobank dataset. Actual resting-state connectome did not survive the permutation test. No significant difference was found $(p > .05, FDR\ corrected)$.

Task Contrast		vs. Actual EMOTION FACES-SHAPES		vs. Resting State Connectome	
		t	*p*	*t*	*p*
Predicted	EMOTION FACES-SHAPES	-	-	-	-
	GAMBLING REWARD	-	-	-	-
	LANGUAGE MATH-STORY	-	-	-	-
	MOTOR AVG	-	-	2.08	.357
	RELATIONAL REL	-	-	-	-
	SOCIAL TOM-RANDOM	-	-	-	-
	WM 2BK-0BK	-	-	-	-
Actual	Resting State Connectome	-	-		

Supplementary Table 24: GAD-7 prediction. The results of paired t-tests with permutation testing ($P = 1000$) comparing the subjects' overall health performance of predicted task contrast maps, actual EMOTION FACES-SHAPES task contrast maps, and resting-state connectome data from the UK Biobank dataset. Significant differences ($p < .05, FDR\ corrected$) are highlighted in bold. Only synthetic MOTOR AVG and actual resting-state connectome significantly predicted GAD-7 scores.

Site	Sample Size	Sex (F)	Age (μ(σ))	Task	TR/TE (ms)	Flip Angle	Multiband factor	Thickness (mm)	Frames
HCP Young Adult	958	504 (52,6%)	28.66 (σ = 3.71)	Resting State	720/33.01 ms	52°	8	2.0 mm	4800
				Working Memory					405
				Motor					284
				Language					316
				Social Cognition					274
				Relational					232
				Emotion Processing					176
				Gambling					253
HCP Development	637	343 (53,7%)	14.49 (σ = 4.05)	Resting State	800/37 ms	52°	8	2.0 mm	976
				Guessing					280*
				Emotion Processing					178
UK Biobank	20,792	11,214 (53,9%)	60.82 (σ = 7.45)	Resting State	735/39 ms	52°	8	2.4 mm	490
				Emotion Processing					332

Supplementary Table 25: Sample details and functional scan acquisition parameters.
*Only Run 1 PA was used to match the Emotion Processing Task.

Input Layer #	Layer #	Layer Type	Normalization	Activation	Output Shape
-	1	Input Data	-	-	50 x 76 x 93 x 78
1	2	Conv3D	BatchNorm3D	ReLU	64 x 76 x 93 x 78
2	3	MaxPool3D	-	-	64 x 38 x 46 x 39
3	4	Conv3D	BatchNorm3D	ReLU	128 x 38 x 46 x 39
4	5	MaxPool3D	-	-	128 x 19 x 23 x 19
5	6	Conv3D	BatchNorm3D	ReLU	256 x 19 x 23 x 19
6	7	ConvTranspose3D (Upsample)	-	-	128 x 38 x 46 x 38
4	8	Attention + Concat	-	-	256 x 38 x 46 x 39
8	9	Conv3D	BatchNorm3D	ReLU	128 x 38 x 46 x 39
9	10	ConvTranspose3D (Upsample)	-	-	64 x 76 x 92 x 78
2	11	Attention + Concat	-	-	128 x 76 x 93 x 78
11	12	Conv3D	BatchNorm3D	ReLU	64 x 76 x 93 x 78
12	13	Conv3D	-	-	47 x 76 x 93 x 78

Supplementary Table 26: DeepTaskGen Architecture. The table is divided into individual convolutional blocks by horizontal lines. Layers 2-6 form the encoding block, which includes the bottleneck, while layers 6-12 constitute the decoding block. Layer 13 consists of a Conv3D with a kernel size of 1. The parameters for convolutional layers from the encoding and decoding blocks are as follows: kernel size = 3, padding = 1, stride = 1. Skip connections from the encoding blocks are filtered through attention gates before being concatenated with their corresponding decoding blocks. During this concatenation, trilinear interpolation is applied to address any slight dimensional discrepancies between the corresponding encoding and decoding layers. The total number of trainable parameters is 3,447,875.

References

1. Abraham, A. *et al.* Machine learning for neuroimaging with scikit-learn. *Front. Neuroinform.* **8**, (2014).
2. Pruim, R. H. R. *et al.* ICA-AROMA: A robust ICA-based strategy for removing motion artifacts from fMRI data. *NeuroImage* **112**, 267–277 (2015).
3. Dice, L. R. Measures of the Amount of Ecologic Association Between Species. *Ecology* **26**, 297–302 (1945).
4. Ngo, G. H., Khosla, M., Jamison, K., Kuceyeski, A. & Sabuncu, M. R. Predicting individual task contrasts from resting-state functional connectivity using a surface-based convolutional network. *NeuroImage* **248**, 118849 (2022).
5. Finn, E. S. *et al.* Functional connectome fingerprinting: Identifying individuals using patterns of brain connectivity. *Nature Neuroscience* **18**, 1664–1671 (2015).
6. Tavor, I. *et al.* Task-free MRI predicts individual differences in brain activity during task performance. *Science* **352**, 216–220 (2016).
