# Supplementary material for: Generating synthetic task-based brain fingerprints for population neuroscience using deep learning
Source: Commun Biol. 2025 Nov 14;8:1572. doi: 10.1038/s42003-025-09158-6 (PMC12618474; doi:10.1038/s42003-025-09158-6)
Supplement: Supplementary file 4 — Reporting Summary [file 42003_2025_9158_MOESM4_ESM.pdf]

nature portfolio

Corresponding author(s): Emin Serin
Last updated by author(s): Oct 12, 2025

Reporting Summary

Nature Portfolio wishes to improve the reproducibility of the work that we publish. This form provides structure for consistency and transparency in reporting. For further information on Nature Portfolio policies, see our Editorial Policies and the Editorial Policy Checklist.

Statistics

For all statistical analyses, confirm that the following items are present in the figure legend, table legend, main text, or Methods section.

n/a	Confirmed	
☐	☒	The exact sample size (n) for each experimental group/condition, given as a discrete number and unit of measurement
☐	☒	A statement on whether measurements were taken from distinct samples or whether the same sample was measured repeatedly
☐	☒	The statistical test(s) used AND whether they are one- or two-sided<br>*Only common tests should be described solely by name; describe more complex techniques in the Methods section.*
☐	☒	A description of all covariates tested
☒	☐	A description of any assumptions or corrections, such as tests of normality and adjustment for multiple comparisons
☐	☒	A full description of the statistical parameters including central tendency (e.g. means) or other basic estimates (e.g. regression coefficient) AND variation (e.g. standard deviation) or associated estimates of uncertainty (e.g. confidence intervals)
☒	☐	For null hypothesis testing, the test statistic (e.g. F, t, r) with confidence intervals, effect sizes, degrees of freedom and P value noted<br>*Give P values as exact values whenever suitable.*
☒	☐	For Bayesian analysis, information on the choice of priors and Markov chain Monte Carlo settings
☒	☐	For hierarchical and complex designs, identification of the appropriate level for tests and full reporting of outcomes
☐	☒	Estimates of effect sizes (e.g. Cohen's d, Pearson's r), indicating how they were calculated

Our web collection on statistics for biologists contains articles on many of the points above.

Software and code

Policy information about availability of computer code

Data collection	No software used to collect data.
Data analysis	Synthetic task data generations were made using PyTorch (>2.2.0) and PyTorch Lightning (>2.2.0). UMAPs were computed using umap-learn package (0.5.6). Further predictions were done using scikit-learn (1.4.1.post1) and scipy (1.12.0). We visualized the results with seaborn (0.13.2). The detailed list of all packages used are given in https://github.com/eminSerin/deeptaskgen-paper/blob/main/requirements.txt.

For manuscripts utilizing custom algorithms or software that are central to the research but not yet described in published literature, software must be made available to editors and reviewers. We strongly encourage code deposition in a community repository (e.g. GitHub). See the Nature Portfolio guidelines for submitting code & software for further information.

Data

Policy information about availability of data

All manuscripts must include a data availability statement. This statement should provide the following information, where applicable:

- Accession codes, unique identifiers, or web links for publicly available datasets
- A description of any restrictions on data availability
- For clinical datasets or third party data, please ensure that the statement adheres to our policy

The minimally preprocessed HCP Young Adult S1200 (HCP-YA) data can be accessed and downloaded from the following link: https://www.humanconnectome.org/study/hcp-young-adult/document/1200-subjects-data-release. The ICA-based group-averaged parcellations used to construct resting-state connectomes are

available at: https://www.humanconnectome.org/study/hcp-young-adult/document/extensively-processed-fmri-data-documentation. Similarly, preprocessed data for the HCP Development (HCP-D) study can be downloaded from https://www.humanconnectome.org/study/hcp-lifespan-development/data-releases. The UK Biobank (UKB) dataset is publicly available at https://www.ukbiobank.ac.uk/.

Research involving human participants, their data, or biological material

Policy information about studies with human participants or human data. See also policy information about sex, gender (identity/presentation), and sexual orientation and race, ethnicity and racism.

Reporting on sex and gender	We used biological sex as one of demographic variables in our validation studies.
Reporting on race, ethnicity, or other socially relevant groupings	We did not use any information related on race, ethnicity, or other socially relevant groupings in any datasets we used in the study.
Population characteristics	HCP-YA: The final sample includes 958 healthy young adult participants (504 females, mean [SD] age=28.66 [3.71] years)).<br>HCP-D: The final sample includes 637 young children or adolescent participants (340 females, mean [SD] age=14.49 [4.05] years)).<br>UKB: A total number of 20,792 participants (11,214 females, mean [SD] age=60.82 [7.45] years)) were included.
Recruitment	We refer the reader to corresponding links or articles for a detailed information on recruitment: HCP-YA (https://www.humanconnectome.org/study/hcp-young-adult/project-protocol/recruitment); HCP-D (Sommerville et al., 2018); UKB (Sudlow et al., 2015).
Ethics oversight	All participants provided written informed consent statements before participation in the study. The HCP data were acquired using protocols approved by the Washington University institutional review board.

Note that full information on the approval of the study protocol must also be provided in the manuscript.

Field-specific reporting

Please select the one below that is the best fit for your research. If you are not sure, read the appropriate sections before making your selection.

☒ Life sciences ☐ Behavioural & social sciences ☐ Ecological, evolutionary & environmental sciences

For a reference copy of the document with all sections, see nature.com/documents/nr-reporting-summary-flat.pdf

Life sciences study design

All studies must disclose on these points even when the disclosure is negative.

Sample size	We included all available data from the relevant datasets, after applying several exclusion criteria explained below.
Data exclusions	HCP Young Adult 1200 Subject Release consists 1206 healthy young participants Of those participants we used 958 who comprise resting-state and all task fMRI images. 39 subjects who have second scanning session were also used as retest in the study.<br>2nd Release of HCP-Development Lifespan dataset contains 652 healthy participants. In our study, we included 637 participants who completed four resting-state runs and Emotion and Guessing tasks.<br>UK-Biobank offers population based (aiming to include 100,000 participants) multi-modal brain images. We selected 20,792 participants with resting-state and emotion task fMRI images and root mean square (RMS) head motion less than 0.5 mm. For prediction, we used 2076 test participants who contains depression status.
Replication	The results found in HCP-YA dataset were then generalized on separate datasets such as HCP-D and UK-Biobank.
Randomization	NA
Blinding	NA

Reporting for specific materials, systems and methods

We require information from authors about some types of materials, experimental systems and methods used in many studies. Here, indicate whether each material, system or method listed is relevant to your study. If you are not sure if a list item applies to your research, read the appropriate section before selecting a response.

Materials & experimental systems

n/a	Involved in the study
☒	☐ Antibodies
☒	☐ Eukaryotic cell lines
☒	☐ Palaeontology and archaeology
☒	☐ Animals and other organisms
☒	☐ Clinical data
☒	☐ Dual use research of concern
☒	☐ Plants

Methods

n/a	Involved in the study
☒	☐ ChIP-seq
☒	☐ Flow cytometry
☐	☒ MRI-based neuroimaging

Plants

Seed stocks: NA

Novel plant genotypes: NA

Authentication: NA

Magnetic resonance imaging

Experimental design

Design type: Functional MRI (task-based and resting-state)

Design specifications: NA

Behavioral performance measures: NA

Acquisition

Imaging type(s): Functional MRI

Field strength: 3 Tesla

Sequence & imaging parameters: HCP-YA: T1-weighted images (TR=2400 ms, TE=2.14 ms, flip angle=8°, FOV=224 x 224 mm, 0.7 mm isotropic voxels); Functional MRI (TR=720 ms, TE = 33.1 ms, flip angle=52°, multi-band factor = 8, 2.0 mm voxels)
HCP-D: T1-weighted images (TR=2500 ms, TE=1.8/3.6/5.4/7.2 ms, flip angle=8°, FOV=256×240×166 mm, 0.8 mm isotropic voxels); Functional MRI (TR=800 ms, TE = 37 ms, flip angle=52°, multi-band factor = 8, 2.0 mm voxels)
UKB: T1-weighted images (TR=2000 ms, FOV=208x256x256 mm, 1 mm isotropic voxels); Functional MRI (TR=735 ms, TE = 39 ms, flip angle=52°, multi-band factor = 8, 2.4 mm voxels)

Area of acquisition: Whole-brain

Diffusion MRI: ☐ Used ☒ Not used

Preprocessing

Preprocessing software: We refer the reader to the original articles for a detailed preprocessing description: Minimal Preprocessing Pipelines (Glasser et al., 2013). Additionally, we used ICA-AROMA to remove noise on the UKB dataset.

Normalization: We refer the reader to the original articles for a detailed preprocessing description: Minimal Preprocessing Pipelines (Glasser et al., 2013).

Normalization template: We refer the reader to the original articles for a detailed preprocessing description: Minimal Preprocessing Pipelines (Glasser et al., 2013).

Noise and artifact removal: We refer the reader to the original articles for a detailed preprocessing description: Minimal Preprocessing Pipelines (Glasser et al., 2013). On the UKB dataset, ICA-AROMA was used to remove noise, and participants with RMS head motion > 0.5 mm

were excluded.

Volume censoring

NA

Statistical modeling & inference

Model type and settings

Synthetic task-based fMRI contrast generation: We generated synthetic task-based fMRI contrast from voxel-to-ROI resting state connectivity using U-Net based volumetric CNN architecture, DeepTaskGen. On HCP-YA, DeepTaskGen was initially trained for 100 epochs using a batch size of 10, Contrast-Regularized Reconstructive Loss (CR-R) as a loss function, and the Adam optimizer. On HCP-D and UKB, the pre-trained model (on HCP-YA) was then fine-tuned for 50 epochs. During fine-tuning, parameters for the corresponding output layer (i.e., EMOTION FACES-SHAPES) were frozen and the backbone of the model were updated. Performance was assessed through reconstruction accuracy (correlation), Dice AUC, diagonality index and fingerprinting scores (Finn et al., 2015).

Demographics, clinical and cognitive variables prediction: We predicted a series of individuals' measures using L2 regularized linear model. The performance of prediction were evaluated within a 5-fold cross-validation scheme and permutation testing (1000 permutations). Balanced accuracy and Pearson's correlation coefficient were used to quantify prediction performance.

Effect(s) tested

Cliff's delta was used to test effect size.

Specify type of analysis: ☒ Whole brain ☐ ROI-based ☐ Both

Statistic type for inference

(See Eklund et al. 2016)

Paired t-tests with permutation testing (1000 permutations) were used to test statistical value of the results.

Correction

FDR correction across all comparison pairs and task contrasts were applied.

Models & analysis

n/a	Involved in the study
☐	☒ Functional and/or effective connectivity
☒	☐ Graph analysis
☒	☐ Multivariate modeling or predictive analysis

Functional and/or effective connectivity

Voxel-to-ROI connectivity was computed as Pearson's correlation between each voxel's time series and the average signal of the target ROI (Khosla et al., 2019). The target ROIs were derived from 50 components obtained through group-level independent component analysis (ICA) applied to resting-state fMRI time series (Smith et al., 2013). The ICA maps are publicly provided: https://www.humanconnectome.org/study/hcp-young-adult/document/extensively-processed-fmri-data-documentation (HCP1200 Parcellation + Timeseries + Netmats (1003 Subjects)).
